# Single-cell level LasR-mediated quorum sensing response of *Pseudomonas aeruginosa* to pulses of signal molecules

**DOI:** 10.1038/s41598-024-66706-6

**Published:** 2024-07-13

**Authors:** Ágnes Ábrahám, László Dér, Eszter Csákvári, Gaszton Vizsnyiczai, Imre Pap, Rebeka Lukács, Vanda Varga-Zsíros, Krisztina Nagy, Péter Galajda

**Affiliations:** 1 HUN-REN Biological Research Centre, Institute of Biophysics, Temesvári Krt. 62, Szeged, 6726 Hungary; 2 Doctoral School of Multidisciplinary Medical Sciences, University of Szeged, Dóm Tér 9, Szeged, 6720 Hungary; 3 Present Address: Division for Biotechnology, Bay Zoltán Nonprofit Ltd. for Applied Research, Derkovits Fasor 2., Szeged, 6726 Hungary; 4 Present Address: HUN-REN Biological Research Centre, Institute of Biochemistry, Temesvári Krt. 62, Szeged, 6726 Hungary

**Keywords:** Cellular microbiology, Lab-on-a-chip, Microbial ecology, Nanobiotechnology

## Abstract

Quorum sensing (QS) is a communication form between bacteria via small signal molecules that enables global gene regulation as a function of cell density. We applied a microfluidic mother machine to study the kinetics of the QS response of *Pseudomonas aeruginosa* bacteria to additions and withdrawals of signal molecules. We traced the fast buildup and the subsequent considerably slower decay of a population-level and single-cell-level QS response. We applied a mathematical model to explain the results quantitatively. We found significant heterogeneity in QS on the single-cell level, which may result from variations in quorum-controlled gene expression and protein degradation. Heterogeneity correlates with cell lineage history, too. We used single-cell data to define and quantitatively characterize the population-level quorum state. We found that the population-level QS response is well-defined. The buildup of the quorum is fast upon signal molecule addition. At the same time, its decay is much slower following signal withdrawal, and the quorum may be maintained for several hours in the absence of the signal. Furthermore, the quorum sensing response of the population was largely repeatable in subsequent pulses of signal molecules.

## Introduction

Social interactions are ubiquitous and essential in natural microbial ecosystems. Quorum sensing (QS) is a crucial form of bacterial communication^1^, enabling bacteria to orchestrate joint actions and form complex communities.

QS is often viewed as a transition of the population between steady states (quorum-on and quorum-off) characterized by phenotypes corresponding to quorum-controlled gene expression patterns^2–4^, which affect, e.g., bioluminescence, metabolic pathways, motility, biofilm formation, sporulation, and virulence^1,5,6^. The transition is governed by cell density through excreted signal molecules: reaching a threshold in signal concentration leads to regulating a set of genes and transitioning from quorum-off to quorum-on state^4,7,8^. To explore the kinetics of this transition, tracking the population’s state is necessary. Luminescence (either natural, as in *Vibrio* species^9–11^, or due to synthetic constructs^12–14^) is ideal for this as it enables both single-cell and population-level monitoring.

*Pseudomonas aeruginosa*, an opportunistic pathogenic bacterium, harbors two acyl-homoserine lactone-based QS systems, LasI-LasR (LasIR) and RhlI-RhlR (RhlIR)^4,15,16^. LasI catalyzes the production of the signal molecule *N*-3-oxo-dodecanoyl-L-homoserine lactone (3O-C12-HSL), which binds to its cognate receptor LasR. LasR forms a homodimer^17^ that, as a transcription factor, controls the expression of numerous genes. In most *P. aeruginosa* strains, the LasIR and RhlIR systems are hierarchically coupled^16,18,19^; however, exceptions exist. For example, in the *P. aeruginosa* PUPa3 strain, the two systems are not hierarchically arranged^20^.

Exploring the kinetics of the quorum off ↔  on transitions is pivotal in understanding how QS functions. Yet, only a handful of papers focus on the kinetics of these transitions in the LasIR QS system^21–26^, and they mainly deal with the quorum off → on direction (buildup of the quorum). Information on the on → off transition is scarce^8,27–29^. This latter decay of the quorum is an essential process without which cells and populations would remain in the quorum-active state forever. Reversibility of QS transitions also ensures that quorum quenching (inhibition of QS) also works on a QS active population^30^, which offers new strategies for infection treatment.

Furthermore, depending on the conditions, the expression of QS-activated genes is not always advantageous. Bentley and co-workers^31^ showed that the benefit of producing "private goods" (which are retained and used up within the cell) doesn't depend on cell density. However, efficient use of the produced extracellular “public goods” was only demonstrated for high population densities. This shows that transitioning from a quorum-on to a quorum-off state is necessary to adapt to changing conditions (e.g., decreasing cell density).

A variety of mathematical modeling approaches have been applied to QS^32^. For example, deterministic single-cell^33,34^ and population-level models^21,34,35^ were worked out, often considering spatial aspects and biofilms and flow^36–41^. In addition, stochastic^39^ and hybrid models on cell^40^ or population scale^41–43^ were put forward. Models focused on diverse aspects of QS, such as evolutionary and ecological implications^44–47^ or therapeutic possibilities^48–52^. The deterministic model developed by Claussen and co-workers^22^ is based on the molecular mechanism of the LasIR QS signaling pathway of *P. aeruginosa* and describes the kinetics of quorum sensing in a synthetic system that employs a QS reporter plasmid. This model considers the expression and dimerization of LasR, its binding to and activation of its target gene, and the expressed proteins' proteolytic decay and growth-related dilution. The analytical solution of this model fits well with experimental data on the onset of the quorum.

There is a growing need for single-cell level experiments on QS due to the recognized significance of phenotypic variability^53,54^. During the last decade, several pieces of evidence were found showing the stochasticity of QS^53,55–60^. Phenotypic heterogeneity may be sourced from the bistability and stochasticity of gene expression, unequal distribution of proteins during cell division, asymmetrical cell division, or epigenetic modifications^61–68^. Such processes result in cell-to-cell variability of signal production and QS response. Moreover, variation of QS on the cellular level can lead to population-level strategies, such as bet-hedging^56^ or division of labor^57^, that increase the chance of survival in changing environments^61^.

Several works demonstrated the importance of QS for single cells^69,70^. These studies suggest that under certain conditions, the induction of QS can be independent of cell density or the spatial structure of a population. Cell-to-cell variability of QS response and QS state (active or inactive) dependent viability was also demonstrated^70^. Heterogeneous QS responses were found profitable in biofilms, too^71^.

Microfluidics has the potential to mimic natural habitats and changing environments in the laboratory by allowing the precise control of physical and chemical conditions^23,72,73^, even on the scale of single cells^74,75^. For example, the mother machine device is suitable for trapping and observing continuously dividing bacterial cells for hundreds of generations^76^.

Here, we studied the QS response of *P. aeruginosa* cells to pulses of signal molecules (*N*-3-oxo-dodecanoyl-L-homoserine lactone) in a microfluidic mother machine. Transitions between quorum sensing states were observed based on the QS-controlled GFP production of cells. The QS response was quantitatively analyzed on the population and single-cell levels as well. QS (or quorum) off → on and on → off transitions were studied, and a numerical model was applied to the population-level data.

## Results

**Figure 1:**
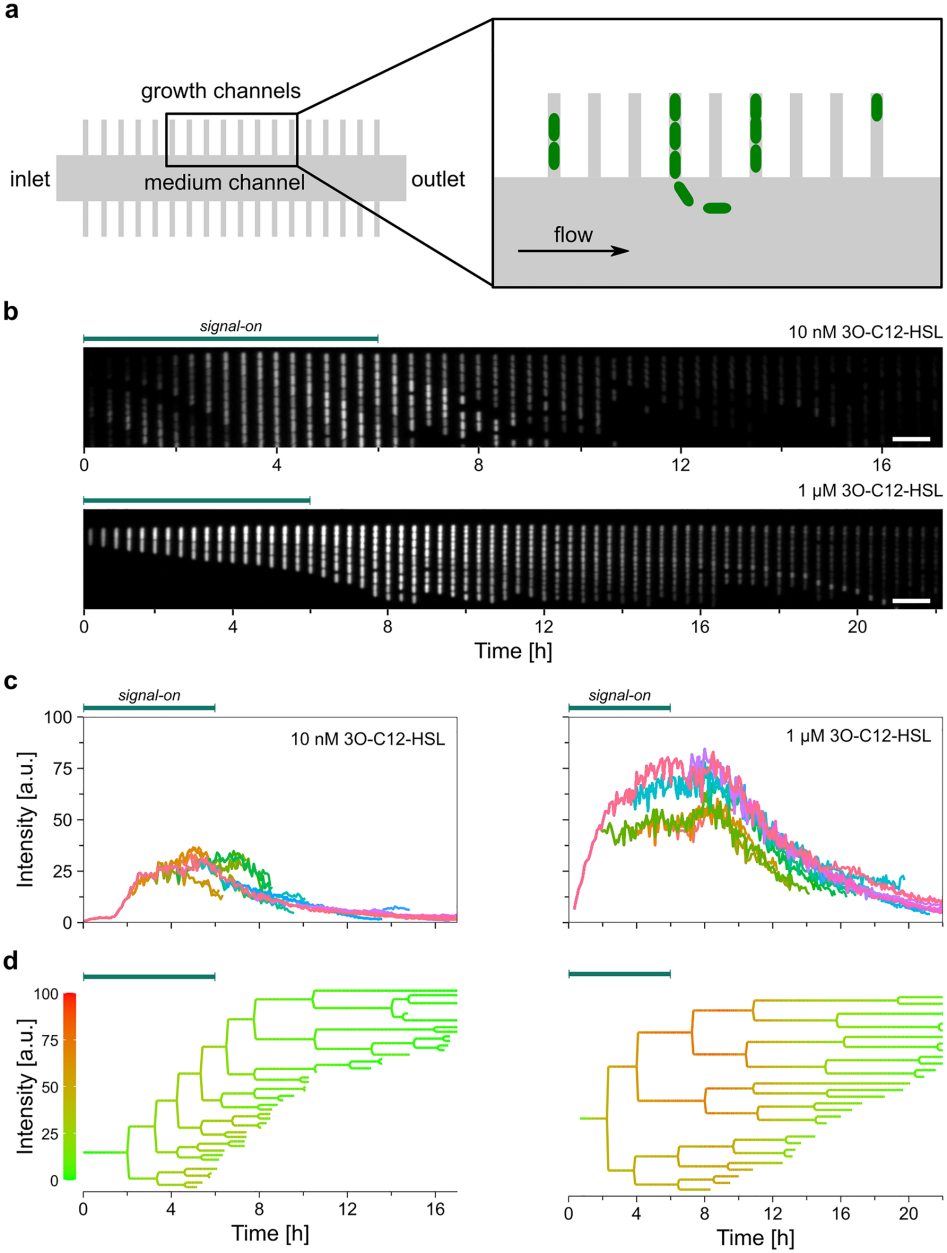
Tracking the quorum sensing behavior of *P. aeruginosa* cells in a microfluidic mother machine device during the application of different concentrations of externally added signal molecules (10 nM and 1 µM 3O-C12-HSL). (**a**) Schematic illustration of the microfluidic device (not scaled). (**b**) Kymograph (i.e., a time series of images) of a single growth channel over the time course of the experiments showing the fluorescence intensity changes of *P. aeruginosa* cells upon adding/removing signal molecules in 10 nM (upper panel) or 1 µM (lower panel) concentrations. The scale bar is 10 µm. Only images taken every 20 min are shown. In addition, see Supplementary Movie 1 and 2. (**c**) Pixel-averaged fluorescence intensity of cells from panel (**b**). Each color represents a new sibling cell that appeared upon division. (**d**) Lineage trees of cells shown in panel (b) with color coding according to pixel-averaged cellular fluorescence intensities.

We applied a microfluidic mother machine device^76^ to expose *lasI*-deficient *P. aeruginosa* PUPa3 cells to an alternating inflow of culture medium with or without signal molecules (3O-C12-HSL) (Fig. 1). In this strain, the feedback coupling between the LasIR and RhlIR quorum sensing circuits is missing^20^, so the functioning of the LasR response regulator is only controlled by externally added 3O-C12-HSL. Furthermore, microfluidic trapping of cells of this strain seemed to be more efficient than the PAO1 strain. Quorum states were traced by monitoring the fluorescence emission of cells due to pKRC12 reporter plasmids coding for GFP under QS control (by using a *lasB* promoter)^77^. Although the plasmid contains a *lasR* copy, LasR within the cells was mostly of genomic origin, and the contribution of the plasmid was negligible (see “Methods” and Supplementary Information).

The experiments contained a 6 h long “signal-on” period, during which cells were continuously exposed to 3O-C12-HSL in a nutrient flow, and a subsequent “signal-off” period when we applied flow without signal molecules. In one set of experiments, we supplied the signal molecules at 10 nM concentration during the "signal-on" period, which is thought to be around or below the QS threshold concentration^77^. In another set, a saturating concentration of 1 µM was used (which signal-producing batch cultures can also achieve^78^). Besides the default "signal-on"-"signal-off" sequence, additional “signal-on” and “signal-off” periods were applied (with a final pattern of on–off-on–off) in some experiments. Changes in fluorescence intensities were monitored using time-lapse microscopy at the single-cell level, along with cell size and division. Cell lineage data were acquired, too. In each kind of experiment, 2444 (10 nM signal) and 7,490 (1 µM signal) cells were analyzed (see “Methods” for details).

### Single-cell level QS response

The schematic drawing of the microfluidic device is shown in Fig. 1a. Cells were harbored in narrow side channels where they lined up with a preserved orientation. This facilitates tracking cell growth, division, and lineage information. Culture media with or without signal molecules were flown in the device through the central channel, from which small molecules diffused in the side channels in seconds. Examples of typical kymographs of single growth channels filled with bacteria and the pixel-averaged fluorescence intensity of the inner cells and their progenies are presented in Fig. 1b,c. The intensity changes show the cellular QS response (following the presence/absence of signal molecules) over the experiment. Figure 1c shows that upon exposure to 3O-C12-HSL, the fluorescence intensities increased and started to level off in 5 h (see Table 1 for the exact values of kinetic parameters yielded by the analysis of all single-cell fluorescence tracks). Withdrawal of the signal molecules resulted in a decrease in fluorescence. Characteristic movies of cells in the growth channels during pulses of signal molecules are also presented (see Supplementary Movie 1 and 2 for 10 nM and 1 μM treatments, respectively).

**Table 1:** Experimental kinetic parameters of the QS response.

3O-C12-HSL concentration	Full data aggregation			Side channel level data aggregation				
	10 nM	1 µM		10 nM	1 µM		*p* value	
		1st pulse	2nd pulse		1st pulse	2nd pulse	Sign. conc	Pulse
Maximal intensity (a.u.)	18.9 ± 12.2	52.3 ± 22.2	55.9 ± 24.0	20.6 ± 10.8	55.7 ± 20.0	62.5 ± 22.2	< 0.001	< 0.001
Fluorescence buildup lag (h)	0.3	0.3	1.8	1.6 ± 0.8	0.4 ± 0.2	2.0 ± 1.0	< 0.001	< 0.001
Fluorescence buildup rate (a.u./h)	2.8	8	8.3	5.3 ± 2.6	10.0 ± 5.3	9.2 ± 5.7	< 0.001	0.185
Fluorescence buildup time (h)	4.7	4.8	4.4	3.0 ± 1.3	5.1 ± 3.1	6.1 ± 3.8	< 0.001	0.018
Fluorescence decay lag (h)	1.3	4.8	5.3	0.4 ± 0.6	3.0 ± 2.8	4.6 ± 3.7	< 0.001	< 0.001
Fluorescence decay rate (a.u./h)	1.5	3.7	3.4	3.3 ± 1.1	4.2 ± 3.5	5.3 ± 5.6	0.001	0.032
Fluorescence decay time (h)	7.7	9.1	9.3	3.5 ± 2.1	10.3 ± 3.0	8.7 ± 3.3	< 0.001	< 0.001

3O-C12-HSL concentration	Full data aggregation			Replicate-level data aggregation				
	10 nM	1 µM		10 nM	1 µM		*p* value	
		1st pulse	2nd pulse		1st pulse	2nd pulse	Sign. conc	Pulse
Quorum buildup lag (h)	2.6	0.7	1.5	2.5 ± 0.7	0.7 ± 0.2	1.0 ± 0.9	0.040	0.576 (0.510)
Quorum buildup rate (1/h)	0.1	0.5	0.4	0.14 ± 0.08	0.5 ± 0.2	0.6 ± 0.1	0.105	0.822 (0.839)
Quorum buildup time (h)	–	1.5	2.1	–	1.5 ± 0.6	1.3 ± 0.3	0.794	0.614 (0.661)
Quorum decay lag (h)	0.8	5.6	5.6	0.2 ± 0.7	6.5 ± 1.6	7.7 ± 2.6	0.012	0.545 (0.270)
Quorum decay rate (1/h)	0.07	0.1	0.07	0.10 ± 0.09	0.12 ± 0.02	0.07 ± 0.02	0.747	0.034 (0.124)
Quorum decay time (h)	–	6.9	10.3	–	6.1 ± 1.5	8.2 ± 2.6	0.080	0.316 (0.164)
Quorum-on duration (h)	0	13.9	13.7	3.5*	14.5 ± 2.2	16.4 ± 3.5	–	0.463 (0.137)

See the “Methods” section and Supplementary Fig. S5c,d for the definitions of the kinetic parameters. Where applicable, mean values and standard deviations are indicated. For the 10 nM signal case, the population-level quorum-on state was only achieved in one experimental replicate. *denotes a quorum-on duration calculated based on this sole replicate. *p*-values were calculated for comparisons of the 10 nM and 1 µM (first pulse) experiments, as well as the 1st and 2nd pulse of the 1 µM experiments (see “Methods”). For comparing the effect of different signal concentrations on the replicate level, the unpaired t-test was used. Both unpaired and paired t-tests were used for the replicate-level analysis of the effect of subsequent signal pulses. *p*-values corresponding to paired t-tests are indicated in parentheses. A linear mixed-effects model was used for the side channel-level analysis. Calculations were made using unpaired data to analyze the effect of signal concentration, and paired data was used to analyze the effect of subsequent signal pulses.

Trends were similar (increase and subsequent decrease in fluorescence) for the 10 nM and 1 µM signal molecule concentration pulses but with some differences. The maximal fluorescence intensity was about three times lower for the 10 nM case presented in Fig. 1c, and there seemed to be a much shorter lag in the fluorescence decrease following the signal withdrawal (see Table 1 for a detailed analysis). In both cases, we observed considerable cell-to-cell variations. Fluorescence intensities and their temporal variations may be markedly different even between the descendants of a single bacterial cell. This is demonstrated in Fig. 1c,d, where some progenies reached higher intensity values after adding 3O-C12-HSL than others (see Supplementary Figs. S1 and S2 for additional fluorescence kinetics data and Supplementary Figs. S3, S4 the corresponding lineage trees).

**Figure 2:**
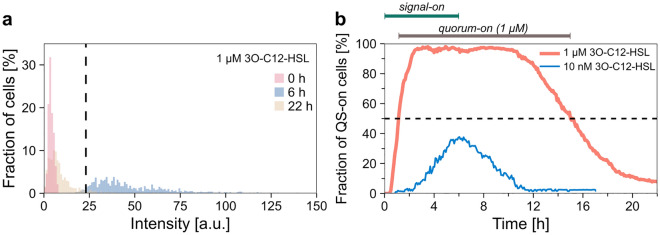
Fluorescence-based determination of quorum states. (**a**) Distribution of the fluorescence intensities of cells within the microfluidic device at characteristic time points for the 1 µM 3O-C12-HSL experiments, respectively: the red histogram corresponds to t_1_ = 0 h (283 cells), the blue one to t_2_ = 6 h (788 cells) and the yellow one to t_3_ = 22 h (675 cells); bin width = 1 a.u. The dashed black line indicates the threshold intensity value (23.1 a.u.) determined based on the 1 µM 3O-C12-HSL data to distinguish QS-on/off states on a single-cell level. (**b**) Fraction of QS-on cells during the experiments. Red and blue lines represent the result from the 1 µM and the 10 nM 3O-C12-HSL experiments, respectively. See Supplementary Fig. S5b for the corresponding cell numbers.

We aggregated all single-cell data from all experimental repeats with the same signal concentration. The distributions of cell-level average fluorescence intensities showed unimodal characteristics (Fig. 2a, Supplementary Fig. S5a). However, the distributions are markedly different at characteristic time points, corresponding to signal-on and signal-off scenarios. Based on the dataset for 1 µM signal concentration, we determined a threshold intensity to assign QS-on and QS-off states to each cell at each time point: cells with intensities over this threshold are said to be in the QS-on state, and those below are in the QS-off state. A threshold intensity of 23.1 a.u. was calculated by maximizing the difference between the number of QS-on cells in the signal-on and signal-off periods in case of saturating (1 µM) signal concentration (see “Methods”).

### Population-level QS response

Based on the above assignment of the QS state of individual cells, the fraction of QS-on cells in the population was calculated, as shown in Fig. 2b. Corresponding kinetic parameters are shown in Table 1. After adding 1 µM 3O-C12-HSL, a short lag (quorum buildup lag of 42 min) preceded a sharp increase in the QS-on cell fraction that took 1.5 h to complete (quorum buildup time). After signal withdrawal, it took 5.6 h for the QS-on fraction to start decreasing gradually (quorum decay lag), finally leveling off in 6.9 h (quorum decay time). When adding signal molecules in 10 nM concentration, the initial increase of the QS-on fraction was preceded by a much longer lag of 2.5 h. Furthermore, the fraction of QS-on cells didn’t quite reach 50% and started to drop shortly (in less than an hour) after signal withdrawal.

We define the quorum state of the population based on the single-cell QS states (determined as described above by applying the threshold intensity). We consider the population to be quorum-on when more than 50% of the cells are in the QS-on cellular state (Fig. 2b). The choice of this threshold is somewhat arbitrary, and different values might be used in various biological scenarios. However, it was shown that some quorum-controlled phenomena require about half the population to be quorum-active^79,80^. Therefore, we proceed with the simple requirement of the majority of QS-on cells. In the 1 µM experiments, populations spent 14 h in the quorum-on state (overlapping with, but considerably longer than, the 6 h signal-on period). For the 10 nM signal concentration, the quorum-on state was only approached. This, in accordance with the work of Riedel et al.^77^, suggests that the threshold signal concentration is around or just above 10 nM.

**Figure 3:**
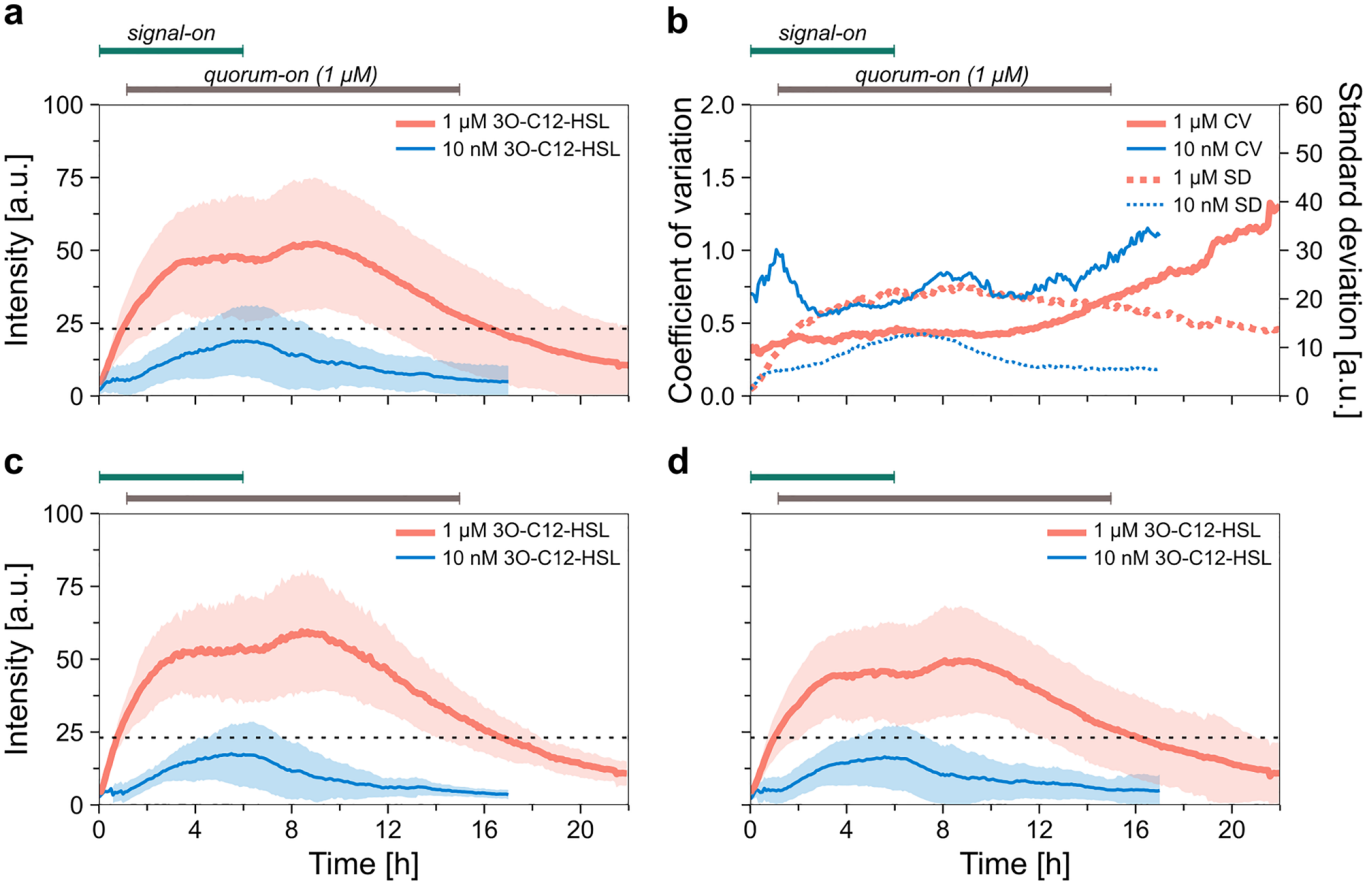
Population-level average fluorescence intensity during pulses of signal molecule. (**a**) Changes in the average fluorescence intensity (together with standard deviation) in case of 10 nM (blue line) or 1 µM (red line) 3O-C12-HSL treatment. The analysis was performed on fully aggregated datasets. See Supplementary Fig. S5b for the corresponding cell numbers. The dashed black line indicates the threshold intensity. (**b**) Coefficient of variation (continuous lines) calculated based on the mean fluorescence intensities and their standard deviations, and standard deviation (dotted lines) during the experiments. Red and blue lines represent the result from the 1 µM and the 10 nM 3O-C12-HSL experiments, respectively. (**c**) Population-level average fluorescence intensity calculated based on the three biological replicates for each signal concentration used. (**d**) Population-level average fluorescence intensity calculated based on side channel-level aggregated data for each signal concentration. Red and blue lines correspond to 1 µM and 10 nM 3O-C12-HSL signal concentrations, respectively. Shaded area shows the standard deviation. The dashed black lines indicate the calculated threshold fluorescence intensity (23.1 a.u.).

We analyzed the population-level average of the fluorescence intensity on three different levels of data aggregation: (1) fully aggregated datasets, (2) side channel-level aggregation, and (3) replicate-level aggregation (see “Methods”). While most of the results we present here are based on the fully aggregated datasets, additional analysis on side channel-level and biological replicate-level aggregated datasets are presented in the Supplementary Information. Figure 3 and Table 1 show the fluorescence kinetics results and the statistical analysis (for the population-level average fluorescence intensities calculated for each biological replicate, see Supplementary Fig. S6.).

The population-average intensity showed regularity in synchrony with the presence of the signal molecule (Fig. 3a). In the case of the 10 nM signal concentration, after an 18 min lag (fluorescence buildup lag, Table 1), there was a steady rise in the average intensity until reaching the maximum. After another short, 1.3 h lag (fluorescence decay lag, Table 1), the withdrawal of the signal molecule led to a steady decrease in intensity, which then leveled off. While some single-cell fluorescence trajectories followed similar dynamics, other cells behaved markedly differently (Fig. 1c, Supplementary Fig. S1), exhibiting diverse fluorescence levels.

In the 1 µM 3O-C12-HSL case, the fluorescence buildup and decay rates and the maximal intensity (8 a.u./h, 3.7 a.u./h, and 52.3 a.u.) were considerably higher than in the 10 nM case (2.8 a.u./h, 1.5 a.u./h, and 18.9 a.u.). While the lag times after signal addition were similar (0.3 h fluorescence buildup lag), the lag following signal withdrawal was considerably longer in the 1 µM signal case (4.8 h versus 1.3 h). When comparing the kinetics of the fluorescence signal to that of the QS-on cell fraction, we see that the transitions are considerably faster in the case of the latter (e.g., 1.5 h quorum buildup time versus 4.8 h fluorescence buildup time, and 6.9 h quorum decay time versus 9.1 h fluorescence decay time in case of 1 µM 3O-C12-HSL, Table 1).

To explore the heterogeneity of the population, we used the standard deviation (SD) and the coefficient of variation (the ratio of the standard deviation and the mean, abbreviated as CV). CV characterizes heterogeneity relative to the average fluorescence intensity, which varied greatly during signal-on and signal-off periods. Figure 3b shows the population-wide SD and CV of single-cell intensities in time. The SD slightly increased and subsequently decreased during a signal-on–off pulse, resembling the shape of the fluorescence curve. The CV increased considerably, demonstrating a high relative cell-to-cell variability in the signal-off phase. A higher CV was observed when 10 nM 3O-C12-HSL was applied (0.7 ± 0.4 compared to 0.4 ± 0.1 for the 1 µM case for the first 16 h), while the SD was lower in these experiments (5.5 ± 3.2 a.u. compared to 13.7 ± 4.6 a.u. for the 1 µM case for the first 16 h).

Cell length, cell cycle length, and cell elongation rate were also tracked during the experiments (see Supplementary Information). While we omit a detailed analysis of these, we note that similar cell lengths and small fluctuations were measured for both signal concentrations (Fig. S7a,b). On the other hand, the cell cycle length was prolonged (and consequently, the elongation rate was reduced) in the high concentration case (1.9 ± 1.2 h cell cycle length in the 10 nM signal case and 3.8 ± 1.6 h the 1 µM case; Fig. S7c–f).

### Numerical model of the population-level QS response

**Figure 4:**
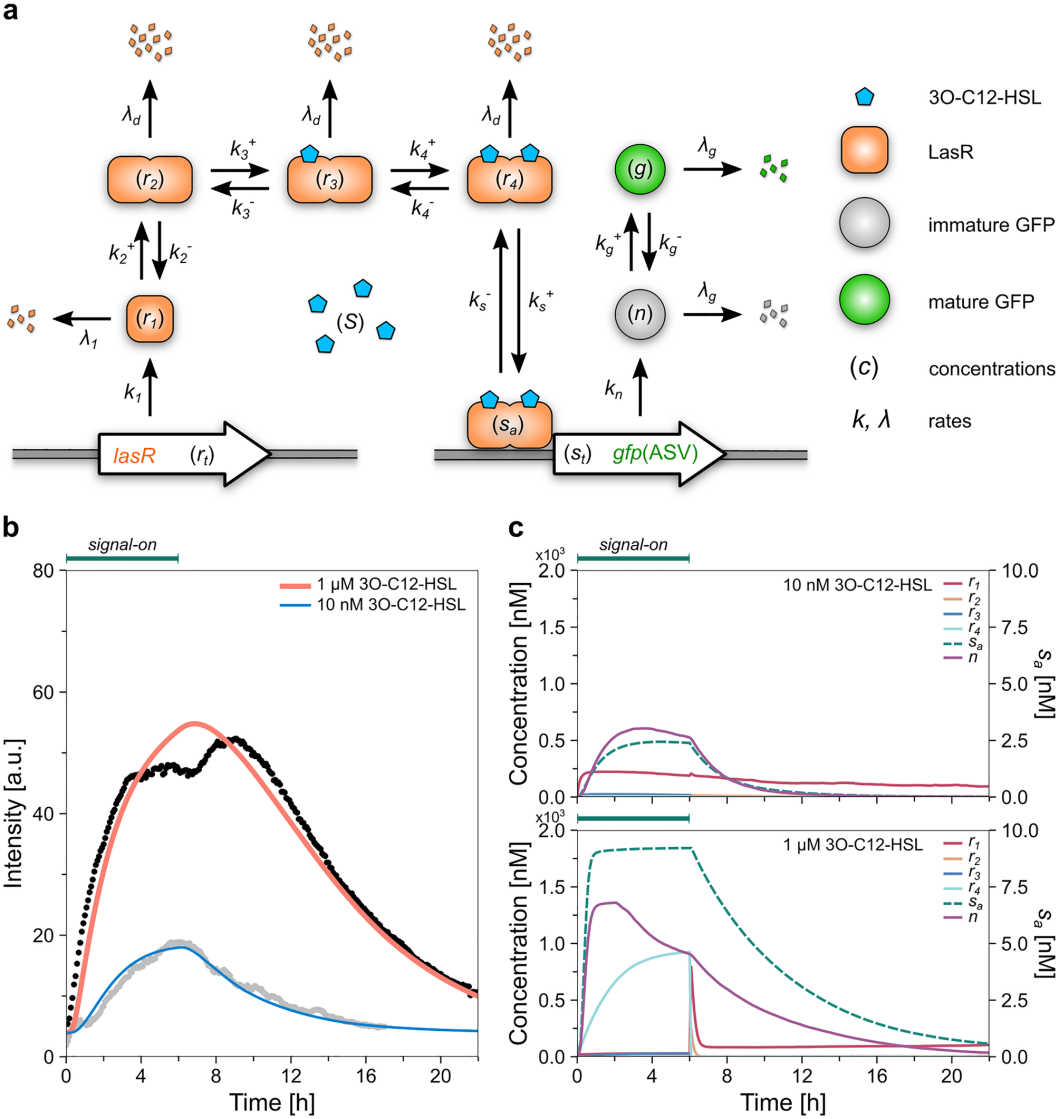
Theoretical model of the population-level QS response. (**a**) Schematic diagram of the functional components used in the model. (**b**) The results of the numerical model (blue line: 10 nM signal molecule concentration, and red line: 1 µM signal molecule concentration) along with the measured average fluorescence intensities (grey dots: 10 nM, black dots: 1 µM). (**c**) Calculated concentrations of molecular species (*r*_*1*_, *r*_*2*_, *r*_*3*_, *r*_*4*_, *n*) included in the model (solid lines), and the concentrations of *gfp* genes with bound LasR-signal complex (*s*_*a*_, dashed line) for both signal molecule concentrations.

To assess the dynamics of the quorum sensing response at the population level, we applied a modified version of the kinetic model by Claussen and co-workers^22^. The model describes the emerging fluorescence signal in batch cultures of *E. coli* harboring a reporter plasmid similar to ours. It considers 13 reactions, including LasR production and dimerization, signal molecule binding to LasR dimers, binding of the LasR-signal complex to DNA, GFP production and maturation, and (proteolytic) protein decay (Fig. 4a). Dilution of proteins due to cell growth is also considered. The beauty of the original model is that an analytical formula was given to describe the kinetics of the emerging (fluorescence) response to the addition of signal molecules. Apart from a scaling factor, this formula contains only measurable parameters, such as maturation and (proteolytic) degradation rates of GFP and the exponential growth rate of the culture. However, we found that the analytical solution gives too fast fluorescence decay rates upon signal molecule withdrawal compared to our experimental results. Therefore, we applied two changes to the model. Instead of a single value for growth rate, we used the time-dependent actual growth rate calculated from our experimental data on cell cycle length (Supplementary Fig. S7a). Furthermore, we considered a linear dependence of *lasR* and *gfp* gene expression rates on growth rate^81–84^ (see “Methods”). Compared to the original model^22^, these modifications resulted in a more accurate modeling of the fluorescence decay upon signal withdrawal.

We fitted this modified model to the measured population average fluorescence data aggregated from the 1 µM and 10 nM 3O-C12-HSL experiments by optimizing the model parameters (Fig. 4b). The fitted model parameters and their values are listed in Table 2. Where they exist, literature data agree well with our results (last column in Table 2). Plasmid (or *gfp*) concentration (*s*_*t*_) was obtained to be 9 nM, in agreement with the literature (5–10 copies/cell^85^). In addition to fitting the GFP degradation rate *λ*_*g*_, we experimentally measured it in our strain (see Supplementary Information and Supplementary Fig. S8). In these experiments, we transferred cells incubated in a signal-on medium into a PBS medium with no nutrients and measured the fluorescence of the culture in time. Fitting an exponential decay curve to the data measured after this nutrient step-down yielded a value of 0.11 ± 0.01 h^−1^ for *λ*_*g*_, in excellent agreement with the model fitting (0.107 h^−1^).

**Table 2:** Parameters used in the kinetic model.

Parameter	Description	Fitted Value	Literature value
$k_{1} \left( t \right) = a + \frac{{1000 \;{\text{nM }}\;{\text{h}}^{ - 1} - a}}{{0.231 \;{\text{h}}^{ - 1} }}\lambda_{c} \left( t \right)$	Production rate of LasR per *lasR* gene		~ 1000 nM h^−1^ ^22,82,108–111^
*a*	Parameter in the k_1_(t) formula (see above)	349.30 nM h^−1^	This study
*c*	y offset	3.933 a.u	This study
*s*_*t*_	Concentration of *gfp* gene	8.986 nM	5–10 copies/cell^85^
*λ*_*1*_	LasR monomer degradation rate	8.975 h^−1^	~ 20 h^−1^ ^22,110–113^
*λ*_*d*_	LasR dimer degradation rate	0.113 h^−1^	~ 0 h^−1^ ^22,29^
*λ*_*g*_	GFP degradation rate	0.107 h^−1^	0.219 h^−1^ ^99^
*k*_*2*_^*−*^ = *k*_*3*_^*−*^ = *k*_*4*_^*−*^	Off rates of LasR dimers	3758 h^−1^	100 h^−1^–4000 h^−1^ ^111,114^; 1000 h^−1^ ^108–110^
*k*_*2*_^+^	On rate of LasR dimer without signal molecule bound	1.490 nM^−1^ h^−1^	0.5 nM^−1^ h^−1^ ^109–111,115,116^
*k*_*3*_^+^ = *k*_*4*_^+^	On rate of LasR dimer with signal molecule(s) bound	235.019 nM^−1^ h^−1^	100 nM^−1^ h^−1^ ^109–111^
*k*_*s*_^*−*^	Complex-DNA off rate	0.044	This study
*K*_*s*_	*k*_*s*_^*−*^*/k*_*s*_^+^_,_ complex-DNA dissociation constant	1.229 nM	1 nM^110^
*k*_*n*_*(t)* = *k*_*1*_*(t)*	GFP production rate of *gfp* gene		~ 1000 nM h^−1^ ^22^
*k*_*g*_	GFP maturation rate	7.709 h^−1^	~ 1.5 h^−1^ ^22,117^
*A*	Scaling factor	0.002	This study

Symbols and short descriptions of the parameters, their fitted value, and data found in the literature.

This modified model reproduces the quantitative and qualitative features of the measured QS response kinetics in both the signal-on and signal-off periods (Fig. 4b). For example, the curve's rising and decaying parts match well with the data. The model correctly reproduces the intensity maxima and the timing of reaching these. For the 10 nM signal concentration, the model well reproduces the lower overall intensity and the non-saturating kinetics.

The kinetics of molecular species calculated from the model are shown in Fig. 4c. In the case of the 10 nM signal, the concentration of the LasR dimers bound to the DNA (*s*_*a*_) increased slowly in the signal-on phase and reached only a low value: less than third of the binding sites were occupied at the maximum (*s*_*a*_ ≈ *s*_*t*_*/*3.6). The LasR monomer concentration (*r*_*1*_) was maintained at a base level in the signal-off period. When 1 µM 3O-C12-HSL was applied, the concentrations of the LasR dimers bound to the DNA (*s*_*a*_) and the immature GFP (*n*) increased faster than in the 10 nM signal case. During the signal-on period, all the available binding sites on the plasmids quickly became occupied by the LasR dimer (*s*_*a*_ ≈ *s*_*t*_), leading to an intense GFP expression. It seems that the maturation process could not keep up with the expression, hence the accumulation of immature GFP. The LasR monomer level (*r*_*1*_) was maintained on a base level even during the signal-off period.

A sensitivity analysis of the model parameters (Supplementary Fig. S9) shows that at saturating signal levels, the GFP degradation rate (*λ*_*g*_), the gene expression rate (through parameter *a*), and the dissociation rate of the LasR complex from the DNA (*k*_*s*_^*−*^) had the most significant effect on the kinetics. This was most likely because reactions leading to GFP expression quickly maxed out upon signal addition, leading to a short period during which the fluorescence increased. Upon signal withdrawal, the decrease in gene expression, the GFP dilution (by cell division), and degradation became the main processes shaping the fluorescence decay, which persisted for an extended period. However, at low signal levels, the slower fluorescence buildup was emphasized in the kinetics and the model fitting. The transition processes between the various LasR forms, as well as the binding of the regulatory complex to DNA, became more critical.

We used the model to estimate the threshold signal concentration of quorum sensing. We calculated the maximal fluorescence intensity the system would reach at various signal molecule concentrations (0.1–150 nM) in a 24 h signal-on period (Supplementary Fig. S10). The concentration-dependent maximal intensity follows a saturating curve, the midpoint of which is 16.8–21.6 nM (depending on the growth rate used in the calculations; see Supplementary Information for details). This suggests that the 10 nM concentration applied in our experiments is close to the QS threshold. Furthermore, the calculations also demonstrate that the QS response saturates around 100 nM signal concentration, well below the 1 µM we used in some experiments.

### Cell lineage information

In addition to the fluorescence intensities, we identified cell division events and traced cell lineage information. Cell cycle length (Fig S7a) was calculated and used for the model calculation to account for growth-rate-dependent gene expression.

**Figure 5:**
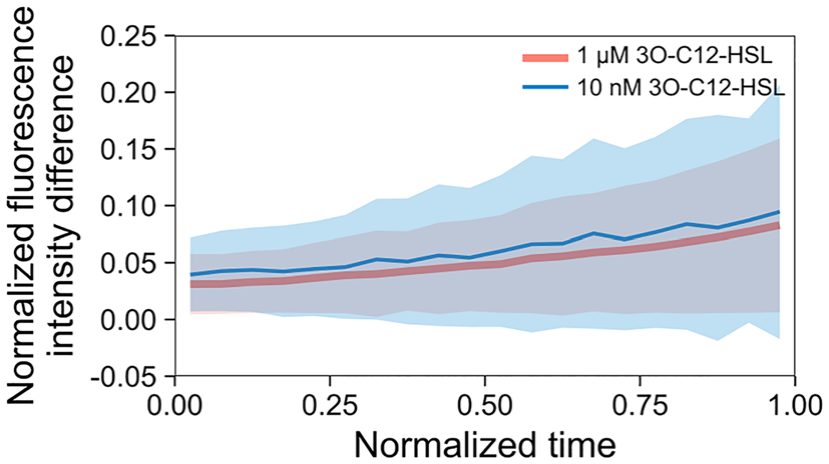
Normalized intensity difference between sibling cells during the cell cycle. Red and blue lines represent the results of the 1 µM and 10 nM 3O-C12-HSL experiments, and the analysis was performed on 1229 and 358 cell pairs, respectively. The shaded area shows the standard deviation. Normalized cell cycle time was calculated as the x coordinate, and data were binned using a bin width of 0.05 along the x-axis.

To gain insight into the emergence of cell-to-cell heterogeneity of the fluorescence intensity, we traced the normalized intensity difference between sibling cells during the cell cycles (Fig. 5, Supplementary Fig. S11). Interestingly, we found a monotonic increase in the normalized intensity difference in time, regardless of the signal concentration applied.

We constructed 167 cell lineage trees from the time-lapse microscopy data (57 lineages from the 10 nM, 110 from the 1 µM 3O-C12-HSL experiments). Examples are presented in Fig. 1d and the Supplementary Information (Supplementary Figs. S3 and S4). These trees are asymmetric and partial due to the continuous dropout of cells from the side channels. Still, an analysis of fluorescence intensity in light of cell lineage information was possible. Here, we define cell lineage distance (CLD) between two cells as the total number of cell divisions separating them from their nearest common ancestor (as in Zhao et al.^86^, see “Methods”). CLD is 2 between pairs of daughter cells, 4 between cousins, etc. CLD is always an even number for pairs of cells from the same generation, while it may be odd for cells from distinct generations.

**Figure 6:**
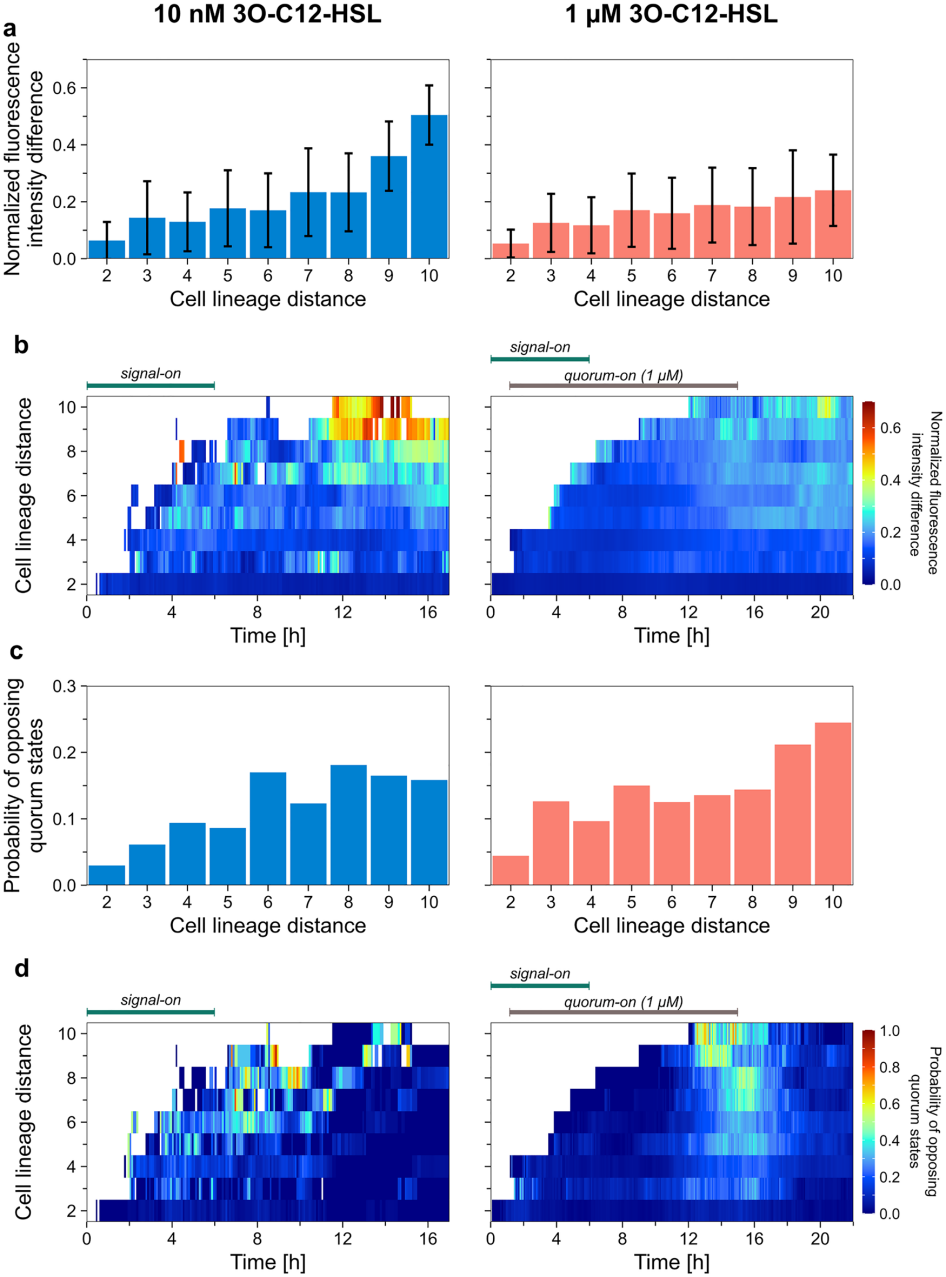
Analysis of phenotypic traits in light of cell lineage information in the case of the 10 nM (left panel) and 1 μM (right panel) signal molecule concentrations. The analysis was performed on fully aggregated datasets. See Supplementary Fig. S13a for the number of cell pairs analyzed. (**a**) Normalized fluorescence intensity data averaged over the time course of the experiment for different cell lineage distance. (**b**) The average normalized fluorescence intensity difference for pairs of cells concurrently present in the device as a function of their cell lineage distance. (**c**) Probability of being in opposite quorum state data averaged over the time course of the experiment for different cell lineage distances. (**d**) Probability of being in opposite QS states for pairs of cells concurrently present in the device as a function of their cell lineage distance.

We analyzed the relative fluorescence intensity difference for all possible pairs of cells concurrently present within the same growth channels in the device as a function of their lineage distance (Fig. 6ab, Supplementary Fig. S12a). Data averaged over the time course of the experiments show that the intensity difference between cell pairs was essentially proportional to CLD (Fig. 6a). The fluorescence level of close relatives was similar, and it differed more for distant relatives. When looking at the intensity differences in time, the most significant deviations were observed in the population-level quorum-off states (i.e., when more than 50% of cells are in the QS-off state) or the late signal-off phase (Fig. 6b).

For the above cell pairs, we also calculated the probability of one cell being in a different QS state than the other (Fig. 6c,d, Supplementary Fig. S12b). This probability also correlates with CLD, meaning that opposite cellular QS states were more frequent for pairs of distant relatives. The probability seems highest at population-level quorum on ↔  off transitions in the 1 µM signal experiments. In the 10 nM experiments, the probability remains high during the signal-on period due to the population closely approaching but not reaching the quorum-on state (Fig. 2b).

Interestingly, there are some linear, diagonal patterns in the temporal change of the probability of opposing quorum states in the low signal experiments in Fig. 6d (left panel). These are due to a small group of closely related cells in the QS-on state when most other cells were QS-off. This results in the fact that at specific time points, e.g., at 14 h, the probability of being in an opposing QS state is low for all except the highest CLD*.*

### Quorum sensing response in repeated pulses of signal molecules

**Figure 7:**
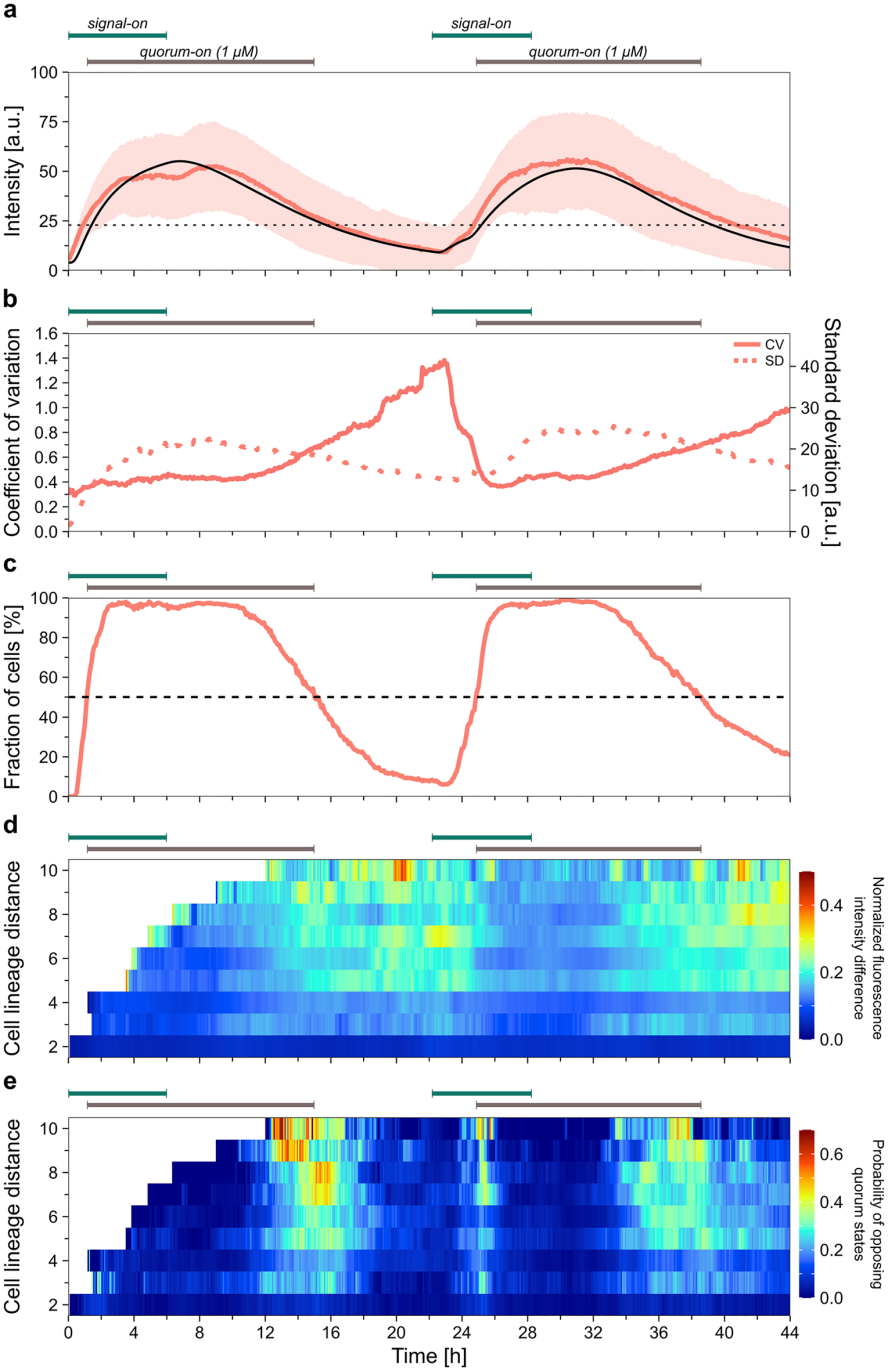
Quorum sensing response of *P. aeruginosa* cells in alternating signal on/off periods, with 1 µM maximal signal molecule concentration. (**a**) The red line shows the population-level average of the fluorescence intensity (the shaded area represents the standard deviation). The dashed black line indicates the threshold intensity (23.1 a.u.). Model calculation of the average fluorescence intensity data is presented by a black line, where the model parameters from Table 1 were used. See Supplementary Fig. S13b for the corresponding cell numbers. (**b**) Coefficient of variation (continuous line) calculated based on the mean fluorescence intensity and its standard deviation, and the standard deviation (dotted line) is presented as a function of time. (**c**) Fraction of QS-on cells within the device during the experiment. (**d**) The average normalized fluorescence intensity difference for pairs of cells concurrently present in the device as a function of their cell lineage distance. See Supplementary Fig. S13c for the number of cell pairs analyzed. (**e**) Probability of being in opposite QS states for pairs of cells concurrently present in the device as a function of their cell lineage distance.

To explore the repeatability of the QS response, we exposed the cells to sequential pulses of signal molecules in saturating concentration (1 µM 3O-C12-HSL). Two 6 h signal-on periods were followed by 16 h signal-off periods (Fig. 7). Cell response was similar to both the first and second signal pulses (Fig. 7a, Table 1), with similar transition rates (8.0 and 8.3 au/h for the buildup, and 3.7 and 3.4 au/h for the decay). The average fluorescence intensity did not vanish entirely in the first signal-off period, but its increase was prompt and fast upon the second signal addition.

We performed model calculations using the parameters determined earlier (Table 2) for two subsequent 1 µM signal pulses (with timings matching the experiments), i.e., no fitting was performed this time. The results of these calculations agree well with the experimental data. According to the model, the molecular species follow similar kinetics in the first and second signal pulses. Cell-to-cell heterogeneity was also comparable during signal-on and signal-off periods; however, the abrupt decrease in CV following the second addition of signal molecules is worth noting (Fig. 7b).

The fraction of QS-on cells within the population reached nearly 100% in both signal waves (Fig. 7c), but the decay was slower in the second signal-off period (0.10 h^−1^ in the first pulse and 0.07 h^−1^ in the second one, Table 1), and not all cells switched back to QS-off states. By the end of the first and second signal-off periods, 9% and 18% of cells remained in the QS-on state, respectively. The normalized fluorescence intensity difference of cell pairs correlated with their cell lineage distance, with higher diversity in both quorum-off periods (Fig. 7d). As seen before, the probability of opposing QS state in randomly selected cell pairs was highest in the quorum on → off transitions. Still, it also peaked briefly in the quorum off → on transitions (Fig. 7e). Overall, the population-level quorum sensing response proved largely repeatable in subsequent pulses of signal molecules.

## Discussion

By applying a microfluidic mother machine device and a fluorescence reporter construct, we gathered data about the QS response of more than 9900 cells to pulses of quorum signals. While previous works focused on the buildup of the quorum response, we also studied how cells and populations return to the quorum inactive state. These data and the deterministic model applied give an exciting insight into the QS response kinetics of *P. aeruginosa* cells.

Quorum sensing is generally considered a population-level phenomenon since, in most scenarios, only a large group of cells can elevate the signal concentration above the threshold. Furthermore, some quorum-controlled behavior, for example, bacterial swarming^87^, emerges only on a population scale. However, the changes in gene expression patterns (and some associated phenotypic changes) in response to the signal appear on the cellular scale. Therefore, cell- and population-level processes strongly intertwine, and the dynamics of QS at the population level can only be understood in detail by analyzing single-cell data.

Determining a population's quorum-sensing state can be challenging, especially in transient conditions. Here we used a quantified phenotypic trait (fluorescence intensity) of single cells to assess the cellular QS state (on or off). Then, we used the statistics of these cellular QS-on/off states to assign quorum-on or -off states to the population. The advantage of this method is that it's quantitative, and even non-binary state assignments may be applied. However, it requires single-cell level measurements, which can be complicated in some scenarios.

Using the quorum-controlled GFP expression as a proxy for the QS state is fair, as the primary action of QS is thought to be to control gene expression patterns. However, we saw that using single-cell fluorescence-based determination of the population quorum state yields faster quorum transitions between quorum states than a population average fluorescence-based method would (faster quorum buildup and decay times compared to the fluorescence buildup and decay times in Table 1).

Although we used a plasmid-based reporter system, due to the low copy number, it imposes a small metabolic burden on the cells^88,89^. Furthermore, it employs a common promoter (*P*_*lasB*_) of 3O-C12-HSL controlled genes^90^ for GFP expression. While the plasmid includes a *lasR* gene, it doesn't contribute to the intercellular LasR pool in the strain we used. These characteristics of the experimental system suggest that similar QS dynamics could be expected for a chromosomal QS system. Mainly, we expect that QS-controlled levels of natural proteins encoded in the chromosome also follow a slow decay upon signal withdrawal, extending the timescale of phenotypic cellular QS (and population-level quorum) on → off transitions in natural ecosystems. The exact kinetics, however, may be different due to, for example, different promoters.

On the population scale, we observed that the response (fluorescence) amplitude was lower in the case of low signal concentration, and the response lag following signal withdrawal was shorter. The transition rates (buildup and decay) were also lower in the low signal case. We consider 10 nM to be slightly below the threshold concentration. We came to the same conclusion after analyzing the number of QS-on cells in the population, which was somewhat below 50% in the 10 nM signal case. Our model calculations also yielded a similar threshold (16–21 nM). This agrees with the findings by Riedel and co-workers^77^, and with the value calculated using our numerical model (16–21 nM). Some threshold concentrations in the literature are in the 100 nM range^78,91^. Our model suggests that parameters affecting the response kinetics, such as protein degradation, gene expression levels, etc., may vary from strain to strain and can depend on growth rate and growth phase, leading to conflicting values in the literature. The 1 µM signal concentration that we also applied is well above saturation. Still, such concentrations were found in signal-producing batch culture^78^. However, experiments at several intermediate signal concentrations would be required to fully explore the effect of the signal concentration on the QS response kinetics.

Signal production and the expression of various QS-controlled genes were shown to vary from cell to cell^53,56–58,60^. Our system lacks the relevant signal-producing enzyme; therefore, we only observe the heterogeneity of the QS response itself. Still, it can be affected by the signal molecule concentration^57^. Here, we applied both near threshold and saturating levels of 3O-C12-HSL. The latter eliminated some of the variability in the signal-sensing pathway, which reduced relative cell-to-cell variations (CV) compared to that measured in the case of lower (threshold) signal concentrations (Fig. 3b). A sensitivity analysis of our model parameters shows that processes directly related to gene-expression play the significant role at high signal concentration, while the transitions between various LasR forms are also at play, and possibly contribute to higher cell-to-cell variations at low signal concentrations.

While the absolute variations of the response level (SD of fluorescence intensity) correlated with the response level, relative cell-to-cell variability (CV) was highest in the late signal-off phase when most cells were in the QS-off state. The numerical model showed that the number of actively expressing QS-controlled genes (*gfp*) decayed slowly in this phase. The parameters most affecting the decay of the response are also related to gene expression and protein degradation. These hint that cell-to-cell changes in active gene copy numbers/expression levels may significantly contribute to heterogeneity in the signal-off phase. At the end of this phase, the number of active *gfp* genes is low, so the stochastic nature of the binding/dissociation of the LasR-signal complex and DNA may result in varying GFP expression leading to a high coefficient of variation of the cellular fluorescence levels. For 10 nM 3O-C12-HSL, there are fewer *gfp* genes expressed even during the signal-on period, which is coupled with higher relative variability in fluorescence (Fig. 3b). Application of cell-based models could further enlighten the details of cell-to-cell variations, something our current model doesn't deal with.

Single-cell fluorescence trajectories show varying QS response kinetics, but the response timing (quorum and fluorescence buildup lag) seems well-defined (see, for example, Figs. 2b, 3, Supplementary Fig. S1, S2 and Table 1). This contradicts those models that suggest a temporal distribution of identical cellular QS histories that results in markedly different population-level dynamics ^43^.

We observed that variations in quorum sensing were higher for distant relatives. In contrast, close relatives demonstrated similar responses (Fig. 6). However, our data show that considerable cell-to-cell differences build up between sibling cells already during a single cell cycle (Fig. 5). Consequently, heterogeneity develops inevitably and fast in a growing population. Interestingly, the difference in QS response between siblings increased monotonically, suggesting that the underlying process is independent of the cell cycle phase. Furthermore, it did not depend on the signal concentration. Slight differences in expression and reaction rates or concentrations of key proteins and degradation rates may lie behind this phenomenon. In line with the above, the difference in fluorescence intensity between cell pairs correlated with their relatedness at all times. While the intensity of sibling cells (CLD = 2) was similar, there was a considerable difference between cousins or more distant relatives (CLD > 2). Furthermore, differences (even for siblings) were more prominent at the late phase of signal-off periods. It has been shown that shared cell lineage history is the primary cause of temporal and spatial cell-to-cell correlations in gene expression levels^92^. Our microfluidic system practically eliminates the possibility of gradient buildup, and our data support the primary importance of cell–cell relatedness in correlated QS-controlled gene expression.

It is important to note that we performed the above analysis on normalized data (relative changes were quantified). However, heterogeneity in the response level (fluorescence) doesn't necessarily translate into variability in biological functioning. For example, the QS state is mainly affected by the response level heterogeneity near the threshold intensity. Still, in several cell cycles, differences may evolve to an extent that influences biological function (Figs. 6d and 7e).

We observed that some cells did not return to the QS-off state after signal withdrawal, which led to the accumulation of QS-on cells at the end of long signal-off periods (Fig. 7c). This was due to the QS-on → off transition being considerably slower in these cells. Despite the slower kinetics, the (population-level) quorum (or cell-level QS) on → off transition inevitably happens upon signal withdrawal. This is important and necessary since it ensures the ability of cells and populations to adapt to conditions that do not favor quorum-induced behaviors. Conversely, the QS-off → on transition was more effective because more than 98% of cells were in the QS-on state in saturating signal conditions. In summary, it is notable that the vast majority (> 80%) of cells get into the same QS state in response to the presence or absence of the signal, which is a prerequisite for an effective population-wide quorum sensing mechanism.

Our results highlight the difference between QS response kinetics in the signal-on and signal-off phases. At high signal concentrations, the onset of QS response is fast. Our model suggests that it is driven by fast forward reactions leading to the formation of dimeric LasR-signal complexes and their activation of GFP expression (Fig. 4c). After signal withdrawal, LasR dimers quickly fall apart into monomers (see the abrupt fall in the concentration of these species on Fig. 4c). However, the dissociation of the LasR-signal regulatory complex from DNA is slow (hence the slow decay of the LasR-signal-DNA complex *s*_*a*_ on Fig. 4c), as well as the decay of the protein under quorum control (GFP in our case). Furthermore, the growth-rate dependence of gene expression rates also slows the decrease in the QS response. An important result of this is the slower decay of the population-level quorum-on, which is still maintained for several hours following signal withdrawal. The sensitivity analysis also demonstrated that at low signal concentrations, the kinetics is shaped by a more delicate balance between the forward and backward transitions between the various LasR forms, as the forward reactions are not maxed out.

To elucidate the data on the QS on → off transition, it is important to note here that the reporter plasmid we employed encodes for an unstable GFP variant (GFP(ASV)). Interestingly, our model calculations and nutrient step-down experiments yielded a half-life of 6.3 h for this GFP variant. This is considerably longer than in the case of other strains, possibly due to a different level of proteolytic activity, and contributes to the slower QS on → off transition.

The fast accumulation of QS-controlled proteins upon signal addition led to an even quicker quorum-off → quorum-on transition on the population level. In contrast, the on → off transition following a signal withdrawal was elongated, due to the slow decrease of the QS controlled protein content. This points towards stabilizing the quorum-on state on the population level (and the QS-on state on the cellular level) and may increase the robustness of quorum sensing. For example, environmental perturbations in space or time may lead to locally or temporally decreased signal molecule concentration (e.g., body fluid flow, biological barriers, or other processes and structures in a host organism). These can be overcome by such a robust system with a stabilized quorum-on (and QS-on) state.

Interference with the QS (e.g., by quorum quenching^93,94^) has been suggested before as a treatment strategy for bacterial infections. The inevitable quorum-on → off transition allows quorum quenching mechanisms to be effective in preventing the formation of a quorum and forcing a quorum-on population into the off state. However, the delay (lag) of this transition should be considered. The quorum-on state and virulence could persist long even after QS signal disruption. On the other hand, if quorum quenching is used as a preventive measure (before an infection), the timing of its application is critical due to the fast quorum off → on transition.

The QS response was quite accurately repeated in subsequent signal-on–off cycles. This sharply contrasts with the single-cell dynamics, where considerable variability was observed (Fig. 1b–c, Supplementary Fig. S1, S2). This suggests that the quorum sensing response in *P. aeruginosa* is much better controlled on the population level than in single cells, similar to *Vibrio fischeri*^60^.

Soil and water are natural habitats for various *Pseudomonas* bacteria. Rain, flood, or watering can transitionally dilute the signal concentration in soil. If these effects are short-term, it may be beneficial for the bacteria to delay the exit from the quorum-on state. Bacteria entering various body fluid compartments during infection in the human body may face similar temporary conditions. For example, the bloodstream or fluid flow in the interstitial compartments may dilute the quorum signal. In the case of lung infections, pulmonary edema or pleural effusion may have similar effects. In such conditions, bacteria may benefit from maintaining the virulent state when the signal concentration is temporarily diluted.

The question arises whether repeated pulses of signals could lead to a population staying continuously in a quorum-on state even between the pulses. While our result suggests such a possibility due to the prolonged decay of the quorum-on state, it remains an open question that further experiments could answer.

Conditions such as pH, nutrient availability, or temperature affect the physiology and metabolism of bacterial cells. Therefore, QS may also be influenced by these parameters. It has been shown that the expression levels of numerous LasR-controlled genes are different at 22 °C or 37°C^95^. This makes sense as the virulence genes seem upregulated at the host temperature. While *lasR* expression is not affected^96,97^, *lasI* is, so a temperature change may influence the QS in signal-producing strains more than in the Δ*lasI* mutant we used.

We included a growth rate dependence in the QS molecular model. This reflects some effects of temperature or nutrient availability, but changes in gene expression or protein degradation are not accounted for due to the fixed model parameters. Additional experiments are needed to explore such details.

While the Rhl and Las systems are shown to be decoupled in the presently used PUPa3 strain^20^, the interplay between the two systems may affect the LasIR QS in other strains, resulting in different kinetics.

In this work, we demonstrated the usefulness of a microfluidic mother machine for single-cell level studies of QS in *P. aeruginosa*. We have shown that subsequent pulses of signal molecules induce a series of similar population-level QS responses. However, there is considerable variability on the cellular level. Besides the buildup of the quorum, we characterized the return of the population in the quorum-inactive state upon signal withdrawal. We applied a mathematical model that quantitatively agrees with our experimental data and helps to shed light on the molecular processes that lead to different QS responses in individual cells.

## Methods

### Bacterial strains, media, and growth conditions

*Pseudomonas aeruginosa* PUPa3 Δ*lasI* mutant strain^20,98^ was used for the experiments. The strain was transformed with pKRC12 gfp-based HSL-sensor plasmid (Gm^R^; pBBR1MCS-5 carrying P_*lasB*_*–gfp*(ASV) P_*lac*_*–lasR*^77^) that is responsive to *N*-(3-oxododecanoyl)-L-homoserine lactone (3O-C12-HSL) and coding an unstable variant of GFP protein (GFP(ASV)^99^). This strain cannot produce the signal molecule 3O-C12-HSL but can sense and respond to it (by turning its quorum system on). Due to the reporter plasmid, the level of GFP expression of the cells gives us information on the quorum state on a single-cell level. We use the term QS-on/off state throughout the manuscript to describe the quorum state of single cells. Quorum-on and quorum-off terms describe the quorum state of the whole population within the device.

According to our control experiments (see *Supplementary Information*), the plasmid's contribution to the LasR expression in the cells is negligible due to the ineffectiveness of the *lac* promoter in *P. aeruginosa*^100^. As described in the *Supplementary Information* (and shown in Fig. S14), no fluorescence (GFP expression) was detected in a plasmid carrying Δ*lasR* mutant even in saturating signal molecule concentrations, which demonstrates that the plasmid doesn’t contribute to the intercellular LasR pool.

*N*-(3-Oxododecanoyl)-L-homoserine lactone was purchased in powder form (Sigma-Aldrich, St. Louis, MO, US) and stored at -20 °C. Stock solution (10 mM concentration) was prepared by dissolving in ethyl acetate containing 0.1% acetic acid. The stock solution was stored at -20 °C and further diluted before each experiment.

Single colonies of *P. aeruginosa* were grown overnight in 3 ml lysogeny broth (LB) medium supplemented with antibiotics (50 μg/ml gentamycin and 50 μg/ml kanamycin for the selection of the reporter plasmid and the Δ*lasI* mutation, respectively) at 30 °C in plastic culture tubes in an incubator shaker at 200 rpm. The overnight cultures were diluted by 1:1000 the following morning. Once they reached OD_600_ = 0.1, 10 nM or 1 μM 3O-C12-HSL was added to the media, respectively, to induce QS. The experiment was started when the culture reached OD_600_ = 0.6 (measured in a test tube). 1.5 ml of the bacteria suspension was centrifuged, and the pellet was resuspended in 300 μl of “*signal-off*” medium. The microfluidic device was inoculated with the cell suspension using a syringe; then we started to flow the “*signal-off*” medium at 200 μl/h overnight (for about 16 h). The fluorescence time-lapse experiment began with a mother machine device in which the side channels were full of cells in the QS-off state. We switched the syringe to the “*signal-on*” medium and started image acquisition. During the experiments, fluctuating signal-on and signal-off periods were applied corresponding to the presence or absence of 3O-C12-HSL signal molecules.

The compositions of the used media during the experiments are the following:

- “*Signal-off*” medium: LB, 10 mg/ml bovine serum albumin, 50 μg/ml kanamycin, 50 μg/ml gentamycin, 0.1% ethyl acetate, 0.0001% acetic acid
- “*Signal-on*” medium: LB, 10 mg/ml bovine serum albumin, 50 μg/ml kanamycin, 50 μg/ml gentamycin, 10 nM or 1 μM 3O-C12-HSL, 0.1% ethyl acetate, 0.0001% acetic acid

Ethyl acetate and acetic acid were needed to dissolve 3O-C12-HSL. To avoid temporal fluctuations, we also added these compounds to the signal-off medium. Bovine serum albumin was included in the media to decrease unspecific cell adhesion to the microfluidic chip surface. Kanamycin and gentamycin were used to select for genomic deletion and plasmid maintenance, respectively.

### Microfluidic device fabrication and loading of the device

The mother machine microfluidic device was fabricated from polydimethylsiloxane (PDMS, Sylgard 184, Dow Inc., Midland, MI, USA) using soft lithography based on^76^. Negative master molds were created in a two-layer structure on a silicon wafer coated with SU-8 photoresist (Microchem Corp., Westborough, MA, USA). For making the shallow side channels that function as traps for bacteria, an SU8-2002 layer of 0.96 ± 0.22 μm height was spin-coated, and the device's design was written in the resist with a Heidelberg μPG101 micro pattern generator (Heidelberg Instruments GmbH, Heidelberg, Germany). The second SU-8 layer was made of SU-8 2015 at 15 μm height, and only the main flow channel was exposed into this layer. To prevent PDMS from attachment to the SU-8 molds, the molds were silanized using (tridecafluoro-1,1,2,2-tetrahydrooctyl) trichlorosilane (Gelest Inc., Morrisville, PA, USA) under vacuum, overnight. Positive replicas were fabricated by molding the PDMS on the master. The cured PDMS (baked overnight at 40 °C in an oven) was peeled off, cut into pieces, and inlet/outlet holes were punched.

Right before starting an experiment, the PDMS device was covalently bound to a glass coverslip by applying oxygen plasma treatment (29.6 W, 400 mTorr, 45 s, in a Harrick PDC-002 plasma cleaner, Harrick Plasma Inc., Ithaca NY, USA). After the plasma binding, the surface of the channels was treated with PLL-g-PEG (SuSoS AG, Switzerland) in 1 mg/ml concentration for 60 min, then washed with LB. Cells were injected by a syringe, and fluid flow was maintained by a SyringeTwo-SKU 4000 syringe pump (New Era Pump Systems Inc., Farmingdale, NY, USA).

In the final devices, the main channels were 18 µm deep, 100 µm wide, and 20 mm long. Each device had 5000 side channels that were 1 µm deep, 1.4 µm wide, and 25 µm long. Variations of the depth and width of the side channels were less than 0.25 µm and 0.1 µm, respectively. Fast diffusion of nutrients and signal molecules ensures that the signal molecule concentration quickly equilibrates between the main and side channels^76^. Due to the dimension of the channels and the fast diffusion of the signal molecule, no gradient buildup takes place in the device^76^.

While the uniflagellar *Pseudomonas* cells may occasionally swim out of the side channels, we did not experience problems in the analysis due to this. The Δ*lasI* PUPa3 strain we used here is reported to have a lower swimming activity than the wild-type strain^20^, which may have helped here.

### Image acquisition

Fluorescence time-lapse microscopy was used to monitor the division and GFP expression of trapped cells throughout the experiment. The experiment was performed at 30 °C using a Nikon Eclipse Ti-E inverted microscope (Nikon Corp, Tokyo, Japan) equipped with a Prior Lumen 200 Pro excitation lamp (Prior Scientific Instruments Ltd, Cambridge, UK) set at 100% intensity and a cage incubator (Okolab S.r.l., Pozzuoli, Italy). A 40 × Nikon Plan Fluor objective, a GFP fluorescence filter set (49,002-ET-GFP filter set with a 470 nm ± 20 nm excitation and a 525 nm ± 25 nm emission filter; Chroma Technology Corp., Bellows Falls, VT, United States), and a Prior Proscan II motorized stage (Prior Scientific Instruments Ltd, Cambridge, UK) were parts of the microscope setup. Time-lapse imaging was done by an Andor NEO sCMOS camera (Andor Technology Ltd, Belfast, UK) and NIS Elements Ar. Software (Nikon Corp, Tokyo, Japan) was used for image acquisition and microscope control. Images were taken every 5 min. The following camera settings were used: 100 ms exposure time, 4 gain, no binning, rolling shutter, and a bit depth of 11.

We performed 3 independent repeats of the experiments for each condition (10 nM or 1 μM 3O-C12-HSL). 10 nM signal experiments were run for 20 h (6 h signal on and 14 h off period), and 1 μM signal experiments were run for 44 h (6 h signal on, 16 h signal off, 6 h signal on, and 16 h signal off).

### Image and data analysis

Time-series images were analyzed using the BACMMAN (BACteria in Mother Machine ANalyzer) software^101^ integrated into the Fiji environment^102^. Details of this analysis are described in the Supplementary Information (Supplementary Figure S15).

Pixel-averaged cell intensities (mean values of pixels assigned to a particular cell), division times, and cell lineage relations were determined using this software. The data produced in BACMMAN were analyzed in R^103^. A background correction was performed on the raw image intensities (see Supplementary Information for details).

In total, 2444 cells were analyzed in the 10 nM signal case (1294, 567, and 583 in the 3 repeated experiments), and 7490 in the 1 µM signal case (2993, 693, 3804 in the 3 repeated experiments). There were 13–264 (10 nM signal case) and 283–915 (1 µM signal case) cells present at a time in the observation channels of the devices. In total, 57 side channels/lineages were analyzed in the 10 nM experiments (20, 20, and 17 in the three experimental repeats), and 110 side channels/lineages in the 1 µM signal case (30, 12, and 68 in the three experimental repeats). The intensity distribution of cells without QS stimulation in the microfluidic device was compared to the histograms measured on non-stimulated cells from batch cultures. The two histograms showed a good agreement (Supplementary Information, Supplementary Fig. S16).

Three methods for data analysis were used. Fluorescence intensity-based analysis was done on fully aggregated datasets where single-cell data from the three repeats of the same experimental condition were merged. If not indicated otherwise, results from this analysis are presented in the manuscript. Where applicable, repeat-level aggregated datasets were used, too, in which single-cell data from each experimental repeat (results shown in the Supplementary Information) were merged. Finally, an analysis of datasets with side channel level aggregation was also performed. Cell-to-cell variation of fluorescence intensity was quantified by calculating the standard deviation based on single-cell data in fully aggregated datasets. In theory, calculating the combined standard deviation for repeat or side channel level aggregated datasets should give the same result^104^. Instead, we calculated the standard deviations of the means for these two aggregation types to account for repeat-to-repeat or side channel-to-side channel variability. The results of the analyses performed on repeat-level and side channel-level aggregated datasets are shown in the Supplementary Information. Parameters describing the kinetics of the mean fluorescence intensities were derived for each of the three analysis methods and are presented in Table 1. The following definitions were used to calculate these parameters (Supplementary Fig. S5c,d). Fluorescence buildup lag: the time elapsed from the beginning of the signal-on period until the intensity increased by 0.1(*I*_*max*_-*I*_*0*_), where *I*_*max*_ is the global maximum intensity, and *I*_*0*_ is the initial (0 h) intensity. *Fluorescence decay lag*: the time elapsed from the beginning of the signal-off period (at 6 h) until the intensity dropped by 0.1 (*I*_*max−*_-*I*_*final*_), where the final intensity (*I*_*final*_) is measured at 17 h for the 10 nM case and 22 h for the 1 μM case. *Fluorescence buildup rate*: (*I*_*90*_ − *I*_*10*_)/(*t*_*90*_ − *t*_*10*_), where *I*_*90*_ = *I*_*0*_ + 0.9(*I*_*max*_ − *I*_*0*_) and *t*_*90*_ is the time when the intensity reaches or crosses *I*_*90*_ earliest. *I*_*10*_ = *I*_*0*_ + 0.1(*I*_*max*_ − *I*_*0*_) and *t*_*10*_ is the time when the intensity reaches or crosses *I*_*10*_ earliest. The *fluorescence buildup time* is *t*_*90*_-*t*_*10*_. The intensity buildup always started in the signal-on period but most often reached over into the signal-off period. *Fluorescence decay rate*: (*I*^*′*^_*90*_ − *I*^*′*^_*10*_)/(*t*^*′*^_*10*_ − *t*^*′*^_*90*_), where *I*^*′*^_*90*_ = *I*_*final*_ + 0.9(*I*_*max−*_*I*_*final*_). *t*^*′*^_*90*_ is the time when the intensity reaches or crosses *I*^*′*^_*90*_ earliest while *t*^*′*^_*90*_ > *t*_*max*_ (*I(t*_*max*_*)* = *I*_*max*_). *I*^*′*^_*10*_ = *I*_*final*_ + 0.1(*I*_*max*_ − *I*_*final*_), and *t*^*′*^_*10*_ is the time when the intensity reaches or crosses *I*^*′*^_*10*_ earliest while *t*^*′*^_*10*_ > *t*_*max*_. The *fluorescence decay time* is *t*^*′*^_*10*_ − *t*^*′*^_*90*_. The intensity decay was always contained within the signal-off period. The mean and standard deviation for these parameters were calculated for the side channel level aggregated datasets.

*Quorum buildup lag* the time elapsed from the beginning of the signal-on period until the fraction of QS-on cells increased by 0.1(*Q*_*max*_ − *Q*_*0*_), where *Q*_*max*_ is the global maximum QS-on fraction, and *Q*_*0*_ is the initial (0 h) fraction. *Quorum decay lag*: the time elapsed from the beginning of the signal-off period (at 6 h) until the QS-on fraction dropped by 0.1(*Q*_*max*_ − *Q*_*final*_ ), where the final QS-on fraction (*Q*_*final*_) is measured at 17 h for the 10 nM case and 22 h for the 1 μM case. *Quorum buildup rate*: (*Q*_*90*_ − *Q*_*10*_)/(*t*_*q90*_ − *t*_*q10*_), where *Q*_*90*_ = *Q*_*0*_ + 0.9(*Q*_*max*_ − *Q*_*0*_) and *t*_*q90*_ is the time when the QS-on fraction reaches or crosses *Q*_*90*_ earliest. *Q*_*10*_ = *Q*_*0*_ + 0.1(*Q*_*max*_ − *Q*_*0*_) and *t*_*q10*_ is the time when the QS-on fraction reaches or crosses *Q*_*10*_ earliest. The *quorum buildup time* is *t*_*q90*_* − t*_*q10*_. The quorum buildup always started in the signal-on period but most often reached into the signal-off period. *Quorum decay rate*: (*Q*^*′*^_*90*_ − *Q*^*′*^_*10*_)/(*t*^*′*^_*q10*_ − *t*^*′*^_*q90*_), where *Q*^*′*^_*90*_ = *Q*_*final*_ + 0.9(*Q*_*max*_ − *Q*_*final*_). *t*^*′*^_*q90*_ is the time when the QS-on fraction reaches or crosses *Q*^*′*^_*90*_ earliest while *t*^*′*^_*q90*_ > *t*_*qmax*_ (*Q(t*_*qmax*_*)* = *Q*_*max*_). *Q*^*′*^_*10*_ = *Q*_*final*_ + 0.1(*Q*_*max*_ − *Q*_*final*_), and *t*^*′*^_*q10*_ is the time when the QS-on fraction reaches or crosses *Q*^*′*^_*10*_ earliest while *t*^*′*^_*q10*_ > *t*_*qmax*_. The *quorum decay time* is *t*^*′*^_*q10*_ − *t*^*′*^_*q90*_. The QS-on fraction decay was always contained within the signal-off period. *Quorum-on duration*: the time the population spends in the quorum-on state between transitions. The mean and standard deviation for these parameters were calculated for the side channel level aggregated datasets. The significance of quantitative differences was evaluated according to the *p*-values calculated in the R software environment. For the replicate-level analysis independent samples t-test was used for analyzing the effect of the signal concentration, and both paired and independent samples t-tests were used to analyze the effect of subsequent signal pulses. The reason behind the latter was that some but not all cells were present in both pulses. A linear mixed-effects model was used for the side channel-based analysis. Unpaired data were used for analyzing the effect of the signal concentration, and paired data were used to analyze the effect of subsequent signal pulses (since the same side channels were analyzed in both signal pulses). Replicates were considered to contribute to the random effect, and the signal concentration or the subsequent pulses were considered to contribute to the fixed effect. The 'lmerTest' package was used for the linear mixed-effects model calculations in R.

The threshold intensity of the QS-on state was calculated based on the first 22 h of the fully aggregated data from 1 µM experiments by scanning through intensities from 0 to 50 a.u. with 0.1 a.u. steps and determining the proportion of cells above and under the applied intensity values at every time point. The time vs. proportion data was smoothened, and the difference of maximum and minimum proportion values was assigned to each intensity, respectively. The step with the maximum difference was selected as threshold intensity.

Cell cycle length was calculated on a per-cell basis as the time elapsed between subsequent divisions, which were identified and timed by BACMMAN. Only those cells were included in the analysis for which BACMMAN identified both divisions. An experimental time coordinate was assigned to all such data at half-time between divisions. The time-dependent exponential growth rate (*λ*_*c*_*(t)*) was calculated from the cell cycle length and used in the model fitting. For some analysis, the normalized cell cycle time was used as an independent variable to represent the time between two divisions within the cycle. It was calculated per cell as the time elapsed from the previous division divided by the time difference between the previous and next division.

For lineage-based analyses, 167 cell lineages were identified (57 for the 10 nM case and 110 for the 1 µM case). Cell lineage distance between pairs of cells was calculated according to Zhao et al.^86^. Namely, the number of divisions between the two cells and their closest common ancestor was counted. Pairwise analysis of cellular intensities was performed at each measurement time point by calculating the relative intensity difference between pairs of cells *|I*_*i*_*—I*_*j*_*| / |I*_*i*_ + *I*_*j*_*|*, where *I*_*i*_ and *I*_*j*_ are the mean fluorescence intensities of cell *i* and cell *j*. This formula was used to calculate the normalized intensity difference between sibling cells (Fig. 6) and more distant relatives (Figs. 7 and 8). Normalized fluorescence intensity data was averaged over the time course of the experiments (Fig. 7) for CLD values from 2 to 10. The number of cell pairs used for these calculations are the following in the case of the 10 nM signal concentration: 13,232, 2565, 14,624, 5546, 13,697, 4352, 5238, 1821, 616 for CLD 2–10, respectively. The number of cell pairs used in the case of the 1 μM signal concentration are 81,745, 22,792, 105,196, 51,177, 96,718, 56,239, 45,666, 18,832, and 5137 for increasing CLD from 2 to10, respectively.

Plots were produced with the ggplot2 R package^105^.

### Mathematical modeling

We employed a modified version of the model by Claussen et al.^22^. A graphical representation of the model is shown in Fig. 4. The model considers LasR production (with a rate of *k*_*1*_) and dimerization (*k*_*2*_), signal molecule binding (*k*_*3*_ and *k*_*4*_), regulator complex binding to DNA (*k*_*s*_), and GFP production (through an immature state of the protein, with rates *k*_*n*_ and *k*_*g*_). All reactions, except LasR production (*k*_*1*_) and proteolytic decay of proteins (*λ*_*1*_, *λ*_*d*_), are reversible. The following differential equations describe the model:

1 $$\frac{\text{d}{r}_{1}(t)}{\text{d}t}={k}_{1}(t){r}_{t}+2{k}_{2}^{-}{r}_{2}(t)-2{k}_{2}^{+}{r}_{1}^{2}(t)-\left({\lambda }_{1}+{\lambda }_{c}\left(t\right)\right){r}_{1}(t)$$

2 $$\frac{\text{d}{r}_{2}(t)}{\text{d}t}={k}_{2}^{+}{r}_{1}^{2}(t)+{k}_{3}^{-}{r}_{3}(t)-2{k}_{3}^{+}{r}_{2}(t)S-\left({k}_{2}^{-}+{\lambda }_{d}+{\lambda }_{c}\left(t\right)\right){r}_{2}(t)$$

3 $$\frac{\text{d}{r}_{3}(t)}{\text{d}t}=2{k}_{3}^{+}{r}_{2}(t)S+2{k}_{4}^{-}{r}_{4}(t)-{k}_{4}^{+}{r}_{3}(t)S-\left({k}_{3}^{-}+{\lambda }_{d}+{\lambda }_{c}\left(t\right)\right){r}_{3}(t)$$

4 $$\frac{\text{d}{r}_{4}(t)}{\text{d}t}={k}_{4}^{+}{r}_{3}(t)S-\left(2{k}_{4}^{-}+{\lambda }_{d}+{\lambda }_{c}\left(t\right)\right){r}_{4}(t)$$

5 $$\frac{\text{d}{s}_{a}(t)}{\text{d}t}={k}_{s}^{+}{r}_{4}(t)\left({s}_{t}-{s}_{a}\left(t\right)\right)-\left({k}_{s}^{-}+{\lambda }_{c}\left(t\right)\right){s}_{a}(t)$$

6 $$\frac{\text{d}n(t)}{\text{d}t}={k}_{n}{s}_{a}-\left({k}_{g}+{\lambda }_{g}+{\lambda }_{c}\left(t\right)\right)n(t)$$

7 $$\frac{\text{d}g(t)}{\text{d}t}={k}_{g}n(t)-\left({\lambda }_{g}+{\lambda }_{c}\left(t\right)\right)g(t)$$

8 $$k_{1} \left( t \right) = k_{n} \left( t \right) = a + \frac{{1000 \;{\text{nM }}\;{\text{h}}^{ - 1} - a}}{{0.231{ }\;{\text{h}}^{ - 1} }}\lambda_{c} \left( t \right)$$

9 $$I(t)={I}_{0}+Ag(t)$$

Here, *S* is the signal concentration (for which we used a step function), *r*_*1*_, *r*_*2*_, *r*_*3,*_ and *r*_*4*_ are the monomeric and dimeric LasR protein concentrations with or without bound signal molecules (Fig. 4a). The *lasR* gene concentration (*r*_*t*_) was fixed as 1 nM (genomic *lasR* concentration). The *gfp* gene concentration is denoted by *s*_*t*_ (which is the pKRC12 plasmid concentration), and the concentration of the “active” (DNA bound) Las regulator complex is *s*_*a*_. The (proteolytic) decay rates of the monomeric, dimeric LasR are *λ*_*1*_ and *λ*_*d*_, respectively. We apply the same decay rate *λ*_*g*_ for immature and mature GFP. At each time point, we derive the exponential growth rate *λ*_*c*_*(t)* from the measured cell cycle length *τ*_*d*_*(t)* as *λ*_*c*_*(t)* = ln*2* / *τ*_*d*_*(t)*. In our version of the model, the rates of gene expressions (*k*_*1*_ and *k*_*n*_) depend linearly on the growth rate according to Eq. (8), which contains a single parameter, *a*. In the case of fast-growing cells (*τ*_*d*_ ≈ 3 h), this formula yields rates of gene expressions that match the values used by Claussen and co-workers^22^. Furthermore, the model builds upon the assumption that the proteolytic decay rate of the LasR dimer is not affected by signal molecules or DNA binding. Finally, the population average of cell intensities *I* linearly depends on the average GFP concentration g, represented by parameters *I*_*0*_ and *A* (Eq. 9). A sensitivity analysis was performed to explore the effect of changing various model parameters on the fluorescence kinetics (see Supplementary Information).

We used MATLAB R2020b^106^ and created a custom script to solve the differential equations and perform fittings (using the Curve Fitting Toolbox^107^) to the experimental average fluorescence intensity data on a PC. The scripts are available on GitHub (https://github.com/sumijate/Quorum, version 1.0). Parameters listed in Table 2 were optimized to achieve the best fit to the average fluorescence data in the 0–24 h period of the 10 nM and 1 μM 3O-C12-HSL experiments (Fig. 4b). Model predictions were calculated using the same parameters for the case of the second pulse of 1 μM 3O-C12-HSL (24–48 period in Fig. 7a).

### Supplementary Information

Supplementary Information.

Supplementary Movie 1.

Supplementary Movie 2.

## Data Availability

All data generated or analyzed during this study are included in this published article (and its Supplementary Information files). Raw data are available from the corresponding authors upon reasonable request. A subset of data is available in the BioImage Archive with the ID S-BIAD1158.
