# Supplementary material for: Single-cell level LasR-mediated quorum sensing response of Pseudomonas aeruginosa to pulses of signal molecules
Source: Sci Rep. 2024 Jul 13;14:16181. doi: 10.1038/s41598-024-66706-6 (PMC11246452; doi:10.1038/s41598-024-66706-6)

Supplementary Information for

Single-cell level LasR-mediated quorum sensing response of *Pseudomonas aeruginosa* to pulses of signal molecules

Ágnes Ábrahám, László Dér, Eszter Csákvári, Gaszton Vizsnyiczai, Imre Pap, Rebeka Lukács, Vanda Varga-Zsíros, Krisztina Nagy*, Péter Galajda*

*To whom correspondence should be addressed.
E-mail: galajda.peter@brc.hu; nagy.krisztina@brc.hu

This pdf file includes:
Supplementary Methods
Legends for Supplementary Movies S1-2
Supplementary Figures S1-16

Supplementary Methods

Well-plate assay to determine the lac promoter activity (in the PKRC12 plasmid) in the used P. aeruginosa PUPa3 strain

P. aeruginosa PUPa3 Δ*lasI* and *P. aeruginosa* PUPa3 Δ*lasR* Δ*rhlR* (signal blind) strains were cultured overnight in LB (supplemented with 50 □g/ml kanamycin and gentamycin) until stationary phase in test tubes (30 °C, 200 rpm). The cultures were back-diluted the following morning by 1:1000 and further incubated until their optical density (measured at 600 nm) reached 0.15. Then, two different samples were prepared of each strain: 1) without 3O-C12-HSL, but 0.1 % ethyl acetate, 0.0001 % acetic acid were added; 2) with 1 □M 3O-C12-HSL, 0.1 % ethyl acetate, and 0.0001 % acetic acid. Six replicates were prepared for each sample and strain with a final volume of 100 □l per well. The experiment was carried out with a BioTek Synergy H1 microplate reader (Agilent Technologies, Inc., Santa Clara, CA USA) at 30 °C, using continuous shaking and reading the OD_{600} and fluorescence intensity (excitation 479 nm, emission 520 nm) values in every 5 minutes for 8 hours. LB (supplemented with 0.1 % ethyl acetate and 0.0001 % acetic acid) solution was used as reference and sterility control during the measurement. Fluorescence intensity data were normalized by the measured OD_{600} values (Supplementary Fig. S14).

Well-plate assay to determine GFP degradation

Three colonies of *P. aeruginosa* PUPa3 Δ*lasI* strain were grown overnight in LB (supplemented with 50 □g/ml kanamycin and gentamycin) until stationary phase in test tubes (30 °C, 200 rpm) and back-diluted in the following morning by 1:500. After 5 hours of incubation (OD_{600} reached 0.45-0.6; measured in the test tubes) 1 µM 3-oxo-C12-HSL was added into the media to induce QS, and incubation continued for 3 hours (during which their OD reached 1.15-1.40). The cultures were then centrifuged two times (5 min, 3500 rpm) and resuspended in PBS media (without signal molecules), in which no growth was expected. The optical density (at 600 nm) and fluorescence intensity of each PBS culture were monitored over time in a 96-well plate. Three replicates were prepared of each culture with 100 µl final volume per well. The experiment was carried out with a BioTek Synergy H1 microplate reader (Agilent Technologies, Inc., Santa Clara, CA USA) at 30 °C, using continuous shaking and reading the OD_{600} and fluorescence intensity (excitation 479 nm, emission 520 nm) values every 5 minutes for 2.5 hours. The measured OD did not change much, which indicates a constant cell number over this period (data not shown). Fluorescence data were fitted by exponentials to determine the time constant of the decay, which gives us information on the lifetime of GFP(ASV) in this strain (Supplementary Fig. S8).

Fluorescence microscopy study to determine the base intensity distribution of P. aeruginosa PUPa3 ΔlasI strain without QS stimulation on single-cell level

A control experiment without QS stimulation of *P. aeruginosa* PUPa3 Δ*lasI* strain was performed using bacteria cultured in test tubes. Three colonies of *P. aeruginosa* PUPa3 Δ*lasI* strain were grown in LB (supplemented with 50 □g/ml kanamycin and gentamycin) until stationary phase (30 °C, 200 rpm), and back-diluted in the following morning by 1:1000. As the optical density (at 600 nm) reached 0.15, 0.1 % ethyl acetate and 0.0001 % acetic acid were added into each culture tube. The cell suspensions were further incubated in the shaker incubator for 24 hours. Then, brightfield and fluorescence images of single cells were taken. For this purpose, 1.5 □l volumes of each culture were dropped onto microscope glass slides and covered with coverslips.

A Nikon Eclipse Ti-E inverted microscope (Nikon Corp, Tokyo, Japan) equipped with a Prior Lumen 200 Pro excitation lamp (Prior Scientific Instruments Ltd, Cambridge, UK) set at 100 % intensity was used for imaging. A 40× Nikon Plan Fluor objective, a GFP fluorescence filter set (49002, Chroma Technology Corp., Bellows Falls, VT, United States), and a Prior Proscan II motorized stage (Prior Scientific Instruments Ltd, Cambridge, UK) were parts of the microscope setup. An Andor NEO sCMOS camera (Andor Technology Ltd, Belfast, UK) and NIS Elements Ar. Software (Nikon Corp, Tokyo, Japan) was used for image acquisition and microscope control. The following camera settings were used for fluorescence imaging: no binning, 100 ms exposure time, rolling shutter, 4 gain, and a bit depth of 11.

Microscopy images were analyzed using Fiji. Phase contrast images were thresholded and used to create a mask to find bacteria on the fluorescence images as well. The average fluorescence intensity of each cell was calculated, and a histogram was prepared to show the distribution of the average fluorescence intensity of single cells without the presence of the 3O-C12-HSL signal molecule (Supplementary Fig. S16).

Image analysis using the BACMMAN software

In the "Pre-processing" step in BACMMAN, background noise removal (Backgroundfit option with Sigma factor 1) and cropping to the region of interest were applied. The microchannels along the y-axis were positioned and aligned using the rotation and flip options. On the final pre-processed image, the microchannel dead-end was at the top of the image.

In the next "Processing" step (while pre-filtering options were omitted), microchannel segmentation/tracking of microchannels and bacteria was done. For microchannel segmentation, we used the MicrochannelFluo2D segmentation algorithm, where the first step was to define the length and width of the microchannel and the y-start shift. These were 154 px, 20-30 px, and 5-20 px, respectively. For thresholding, we used Backgroundfit with Sigma factor 1. The filling proportion parameter and the minimal object size were set to 0.05 and 3, respectively.

For the segmentation of bacterial cells, we used the BacteriaFluo segmentation algorithm, in which we chose the Hysteresis_thresholding as a foreground selection method. For background and foreground thresholding, we used Parent_track. The Method selected for these was Backgroundfit with Sigma factor 1 in both cases.

Two parameters affect the segmentation of dividing cells. For the Hessian scale, we used 2, and the Split Threshold was 0.001. Occasional errors in the BACMMAN image analysis (e.g., missensed divisions lasting for single frames) were manually checked and corrected.

An example of bacteria segmentation and tracking by BACMMAN is presented in Supplementary Fig. S15.

Background correction of the raw image data

A background correction was performed on the raw pixel-averaged fluorescence intensities (I) by applying the following formula:

$$I_{corr} = \frac{I - B}{B - D}$$

where B is the average background intensity (measured in the absence of fluorescent bacteria in the vicinity of the mother machine side channels) and D is a camera specific average dark intensity (measured with the camera sensor blocked from all light sources).

Measuring cell length and elongation rate

The population average of cell length and elongation rate was measured on fully aggregated datasets and on biological replicate-level (Supplementary Fig. S7). The parameter "spine length" calculated by the BACMANN software was used as cell length. This parameter is defined as the central line crossing the bacterium from one pole to the other. (Each point of the spine is equidistant from the two closest points of the contour located on each side of the spine.)

Elongation rate is defined as the difference between cell length measured at the beginning and at the end of the cell cycle, divided by the cell cycle length.

Mathematical model to determine the QS threshold concentration

By using different signal molecule concentrations (S) in the mathematical model of the QS response, the theoretical threshold was determined. The concentrations varied from 0 to 150 nM with 0.5 nM step size. Calculations were done using a lower and a higher static growth rate (calculated from the measured cell cycle length at the 6 h timepoint in the experiments with 10 nM (1.3 h) and 1 μM (4.3 h) signal concentration, respectively). The dynamics of the intensity change were calculated for 22 hours for each concentration. The maximum intensity values in the function of the corresponding concentrations are presented in Supplementary Fig. S10. The threshold concentration was determined by finding the midpoint of the curves, which was found to be in the 16.8-21.6 nM range.

Sensitivity analysis of the model parameters

We performed a sensitivity analysis to determine the extent to which the change of one parameter affects the resulting kinetics. For this, we changed the fitted value of the model parameters by ±15 %, one at a time. The original fitted curve and the new curve (calculated using the changed parameter) were compared by calculating the ratio of the residual sum of squares and the total sum of squares (which is practically $1-R^2$). This value is indicated in Supplementary Fig. S9.

Legends for Supplementary movie S1-2

Supplementary Movie S1

P. aeruginosa Δ*lasI* bacteria were treated by 10 nM 3O-C12-HSL signal pulse. The movie was prepared from the time-lapse image series of a selected growth channel during one signal on/off period. Images were taken every 5 minutes.

Supplementary Movie S2

P. aeruginosa Δ*lasI* bacteria were treated by repeated pulses of 1 □M 3O-C12-HSL signal molecules. The movie was prepared from the time-lapse image series of a selected growth channel during two successive signal on/off periods. Images were taken every 5 minutes.

Supplementary Figure S1.

The kinetics of 16 individual cell lines captured for one signal on/off period. The concentration of the signal molecule was 10 nM. Characteristic data were selected from each biological replicate. Plot labels indicate signal concentration, biological repeat, and side channel number. Graphs show the changes in the fluorescence intensity of cells and their progenies. Each color represents a new cell that appears after a division.

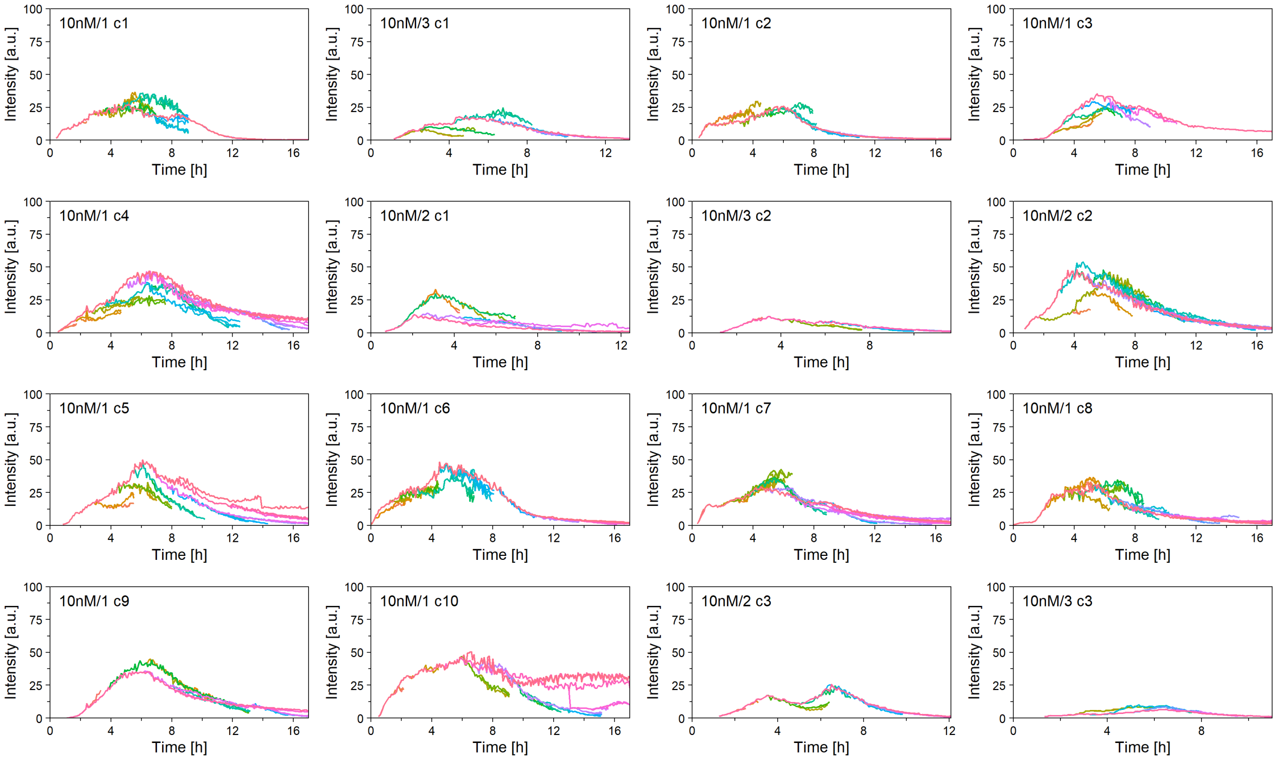

Supplementary Figure S2.

The kinetics of 16 individual cell lines captured for one signal on/off period. The concentration of the signal molecule was 1 µM. Characteristic data were selected from each biological replicate. Plot labels indicate signal concentration, biological repeat, and side channel number. Graphs show the changes in the fluorescence intensity of cells and their progenies. Each color represents a new cell that appears after a division.

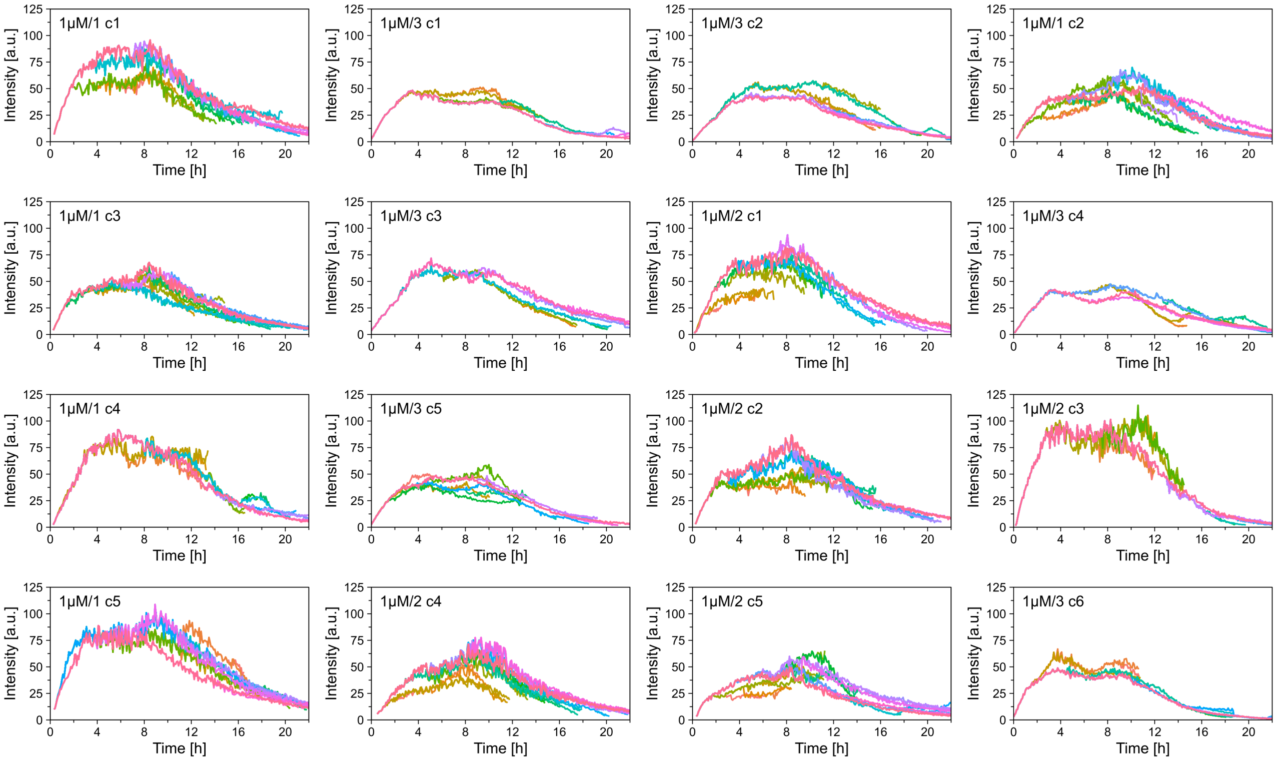

Supplementary Figure S3.

Cell lineage relations together with intensity data of the selected 16 cell lines captured for one signal on/off period. The applied signal molecule concentration was 10 nM. The cell lineage trees always start from the innermost cell within a growth channel that is present from the beginning of the experiment. Data were selected from each biological replicate. Plot labels indicate signal concentration, biological repeat, and side channel number (identical to those present in Supplementary Fig. S1). Changes in the fluorescence intensities are indicated by using a color bar.

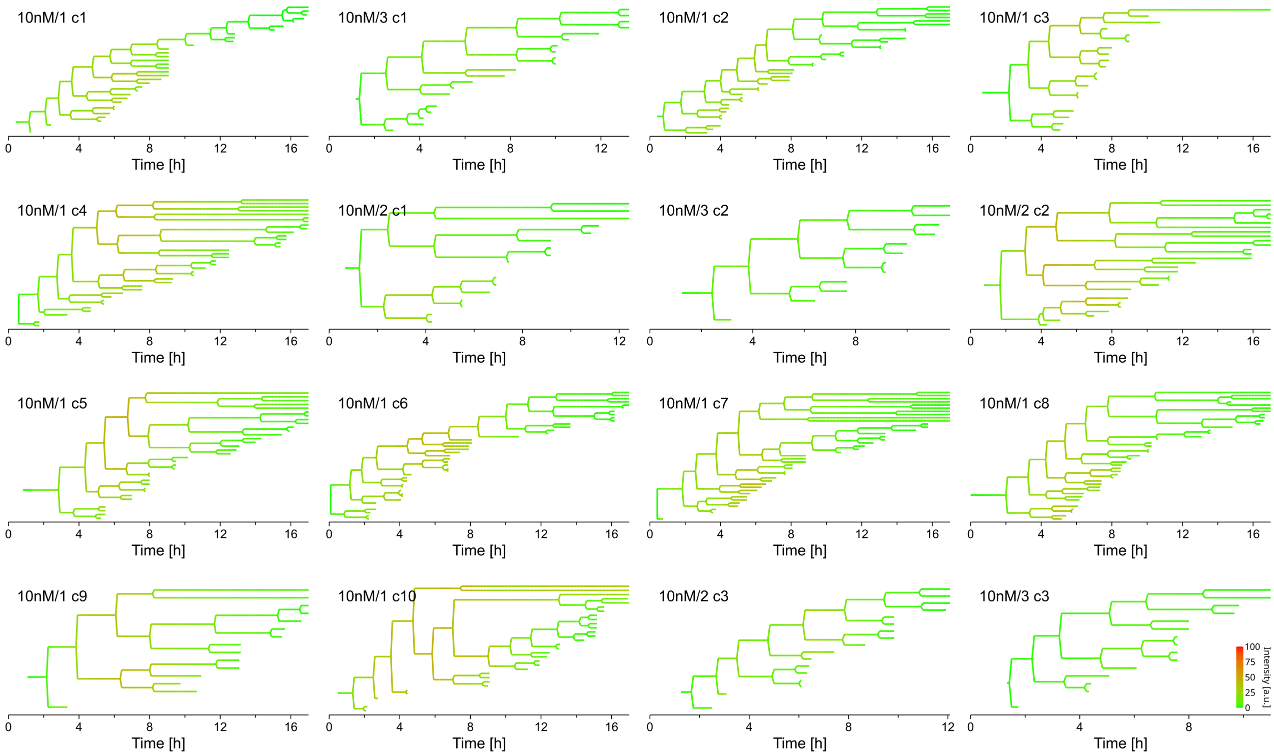

Supplementary Figure S4.

Cell lineage relations together with intensity data of the selected 16 cell lines captured for one signal on/off period. The applied signal molecule concentration was 1 µM. The cell lineage trees always start from the innermost cell within a growth channel that is present from the beginning of the experiment. Data were selected from each biological replicate. Plot labels indicate signal concentration, biological repeat, and side channel number (identical to those present in Supplementary Fig. S2). Changes in the fluorescence intensities are indicated by using a color bar.

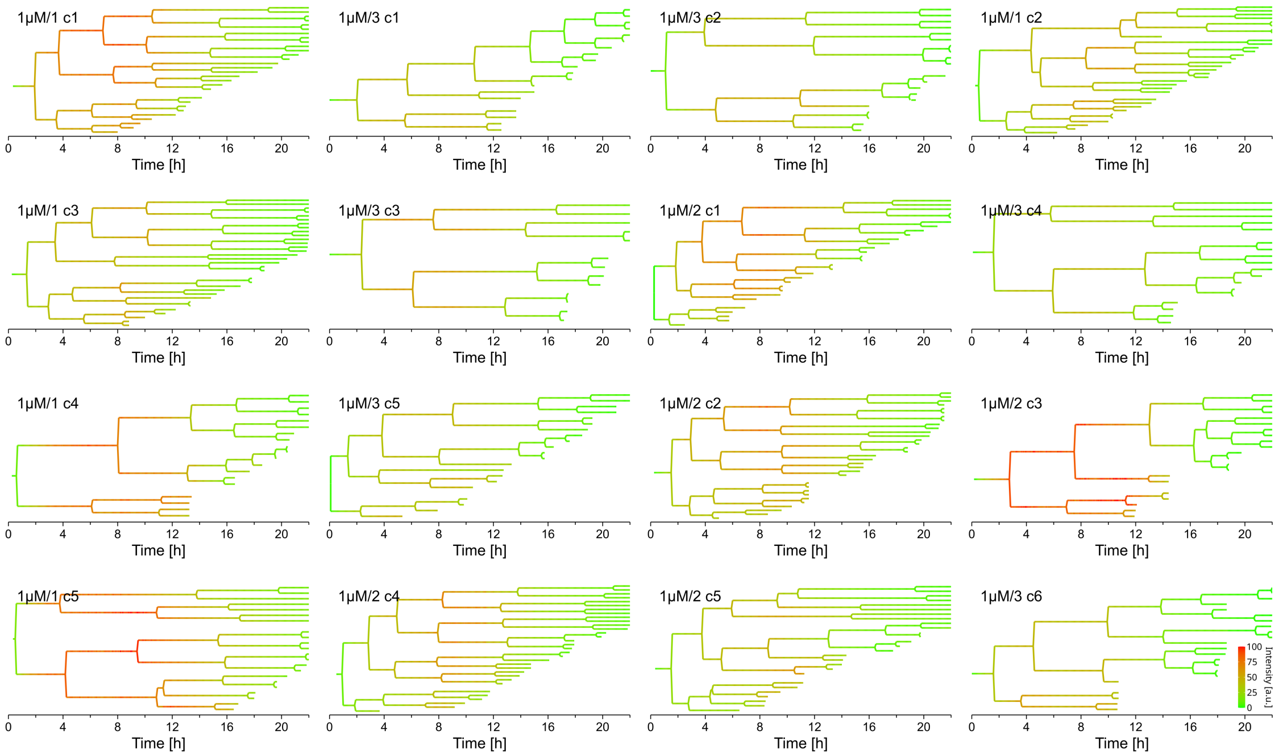

Supplementary Figure S5

Cell-level average fluorescence intensities, cell numbers and derivation of the kinetic parameters. a) Distribution of cell-level average fluorescence intensities in case of 10 nM (left panel) and 1 μM (right panel) signal molecule concentrations. The data analysis was carried out on fully aggregated datasets. Intensity bin width is 5 a.u., time bin width is 15 min. Light grey dashed line indicates the threshold intensity (23.1 a.u.). The fraction of cells with certain intensity level at a time point is color coded. b) Aggregated cell number over the time course of the experiments. Blue and red lines represent the datasets of experiments applying 10 nM or 1 μM signal concentration treatments, respectively. c) and d) Illustration of quantities used to derive kinetic parameters.

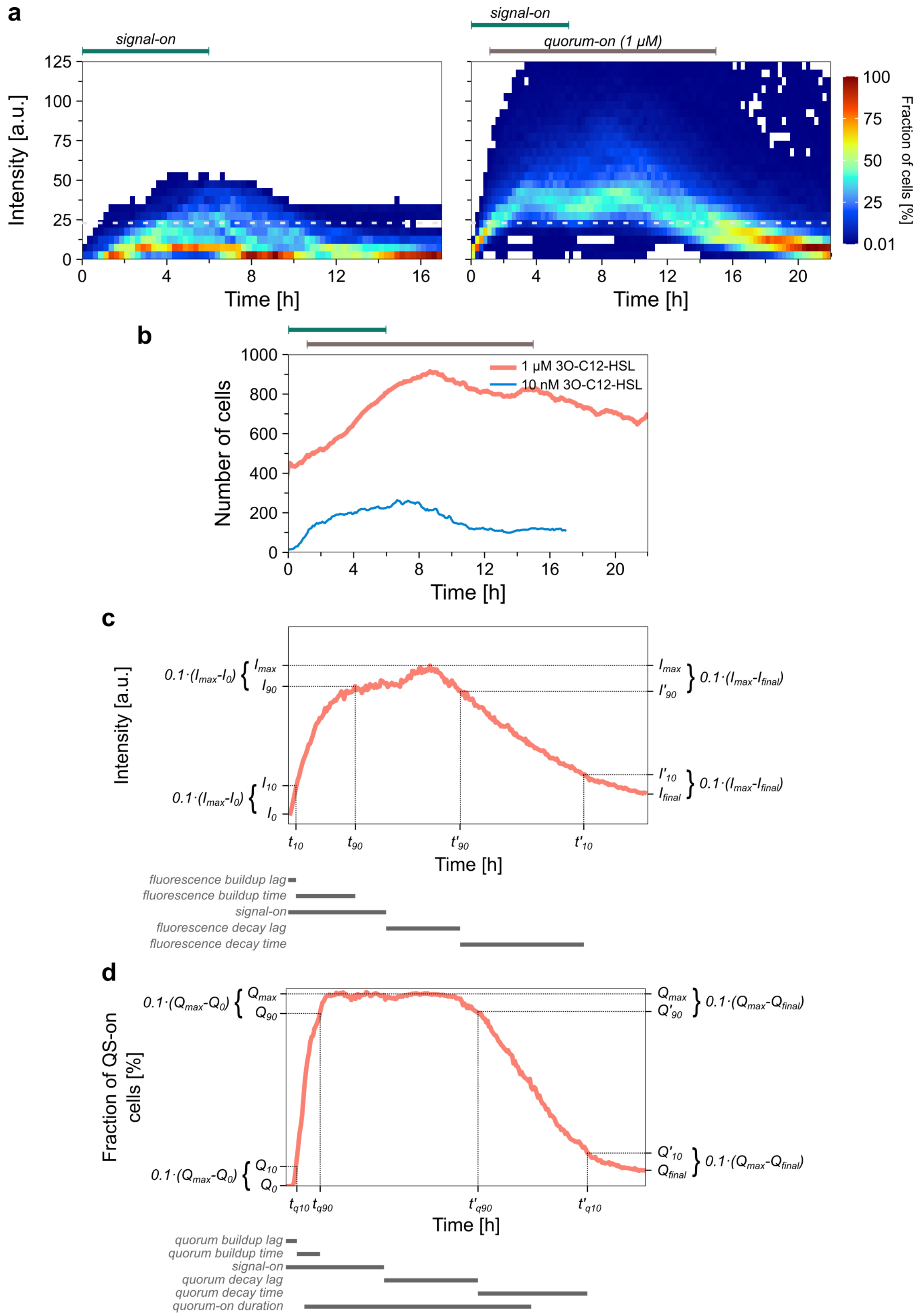

Supplementary Figure S6.
Population-level average fluorescence intensities calculated for each biological replicate. The left panel shows the three replicates of 10 nM 3O-C12-HSL signal concentration (blue curves and the shaded area represent the mean fluorescence intensity and its standard deviation over time). The right panel shows the three replicates of 1 µM 3O-C12-HSL signal concentration (red curves and the shaded area represent the mean fluorescence intensity and its standard deviation over time). The black dashed line corresponds to the threshold intensity (23.1 a.u.).

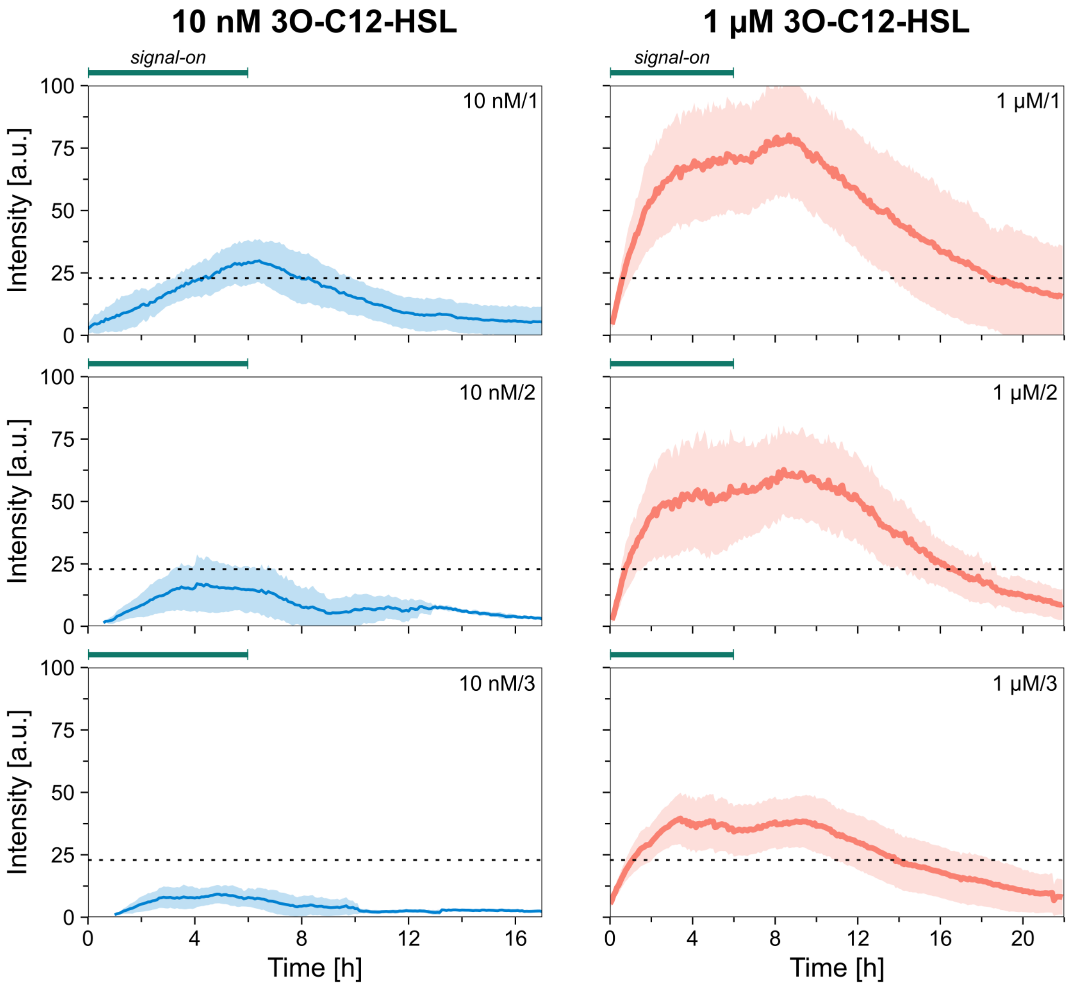

Supplementary Figure S7

Experimental data for the average cell cycle length, cell length and elongation rate in case of using 10 nM (blue line) or 1 µM (red line) 3O-C12-HSL concentration. a, c and e panels correspond to data calculated by using fully aggregated datasets, while b, d and f panels represents averages of biological replicates. Continuous lines show the average values and the shaded area is the standard deviation.

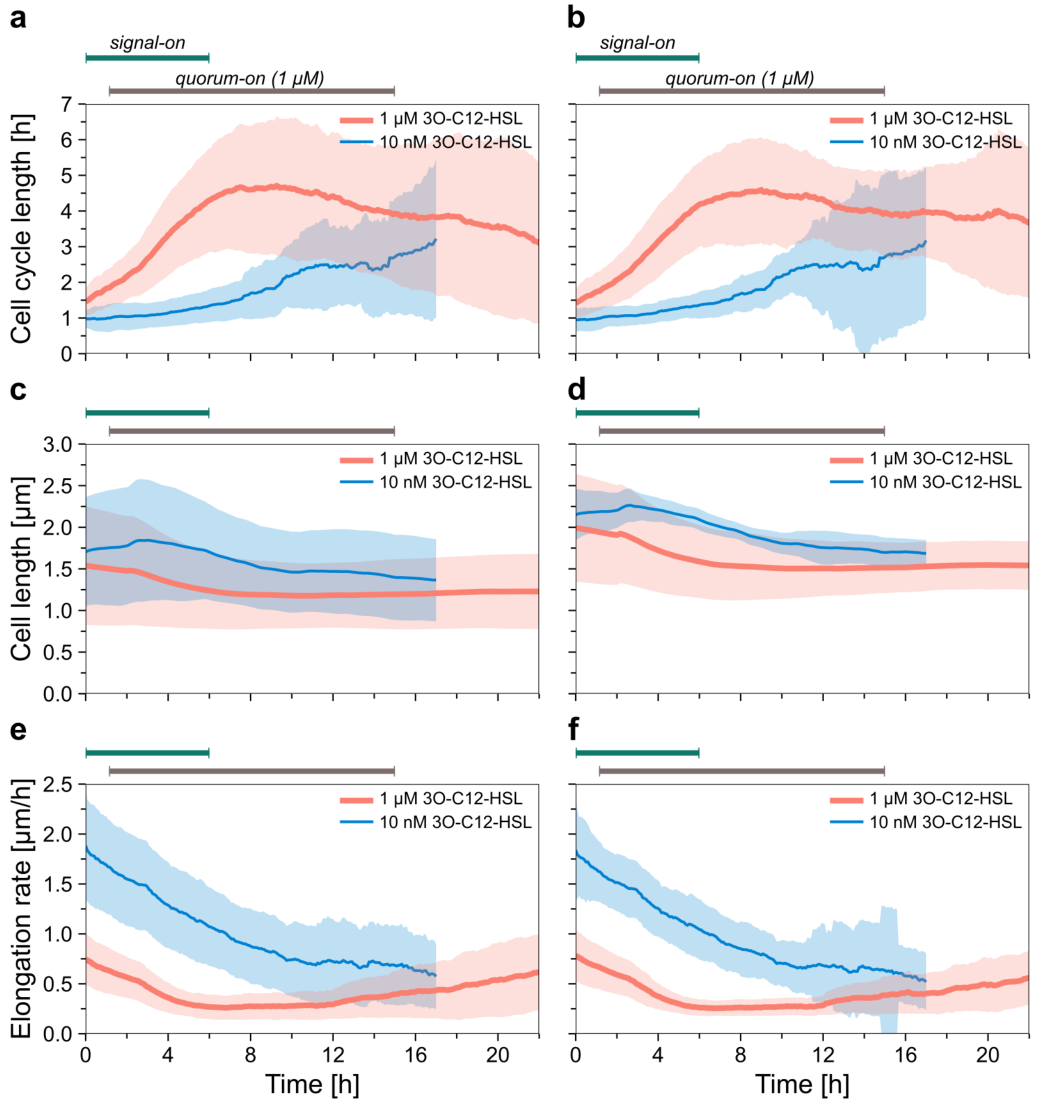

Supplementary Figure S8

96-well plate study to determine GFP degradation in *Pseudomonas aeruginosa* PUPa3 Δ*lasI* strain. The decrease of the fluorescence signal was measured upon transferring the bacteria culture from LB to PBS medium (as a nutrient step-down). Intensity was measured every 5 minutes for 2.5 hours. Three independent colonies, each of them in three replicates, were used (blue, red, and green data sets in the figure). Data were fitted by exponentials: λ_g is the GFP degradation rate.

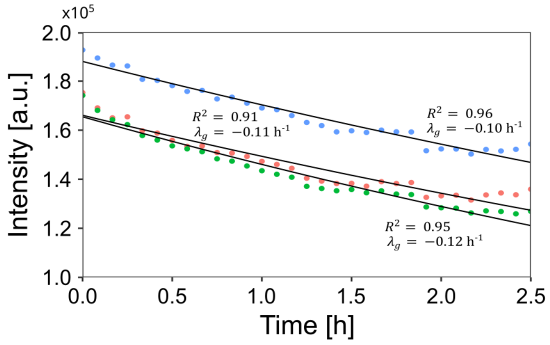

Supplementary Figure S9

Sensitivity analysis of the model parameters. The fitted values of the model parameters were changed by ±15 % one at a time, and the ratio of the residual sum of squares and the total sum of squares was calculated. Results for a -15% and a +15% parameter change are shown in red and turquoise, respectively.

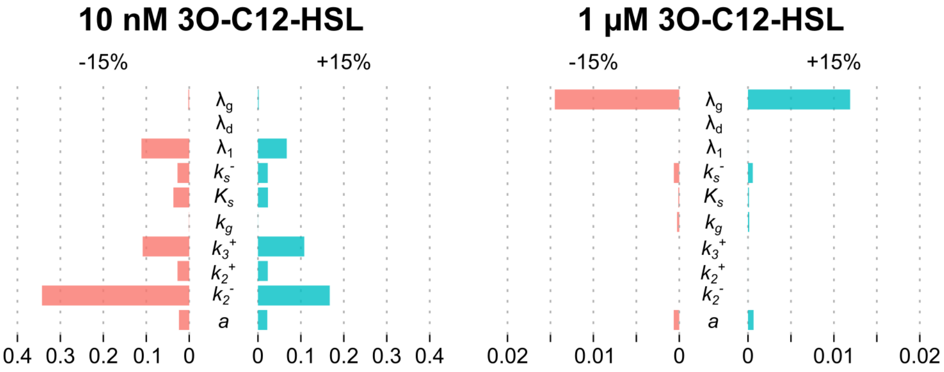

Supplementary Figure S10.

Model-based estimation of threshold concentration. The graph shows the maximum normalized intensity as a function of signal molecule concentration. The blue and red curves were calculated by applying lower and higher static growth rates in the model (for details, see Supplementary Methods). The threshold concentration was found to be between 16.8-21.6 nM indicated by the thin and thick dashed black lines, respectively.

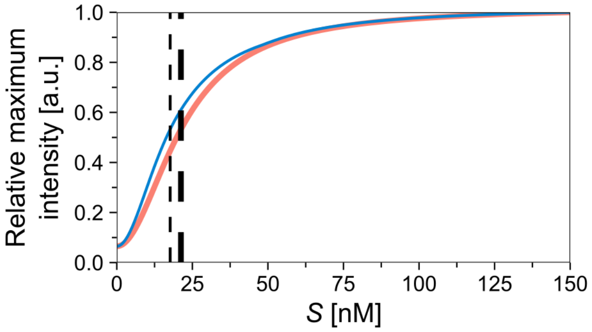

Supplementary Figure S11.

Replica-based analysis of normalized intensity difference between sibling cells during the cell cycle. The left panel shows the three replicates of 10 nM 3O-C12-HSL signal concentration (blue lines and the shaded area represent the average normalized intensity difference and its standard deviation over time). The right panel shows the three replicates of 1 μM 3O-C12-HSL signal concentration (red lines and the shaded area represent the average normalized intensity difference and its standard deviation over time). Normalized cell cycle time was calculated as the x coordinate, and data were binned using a bin width of 0.05 along the x-axis.

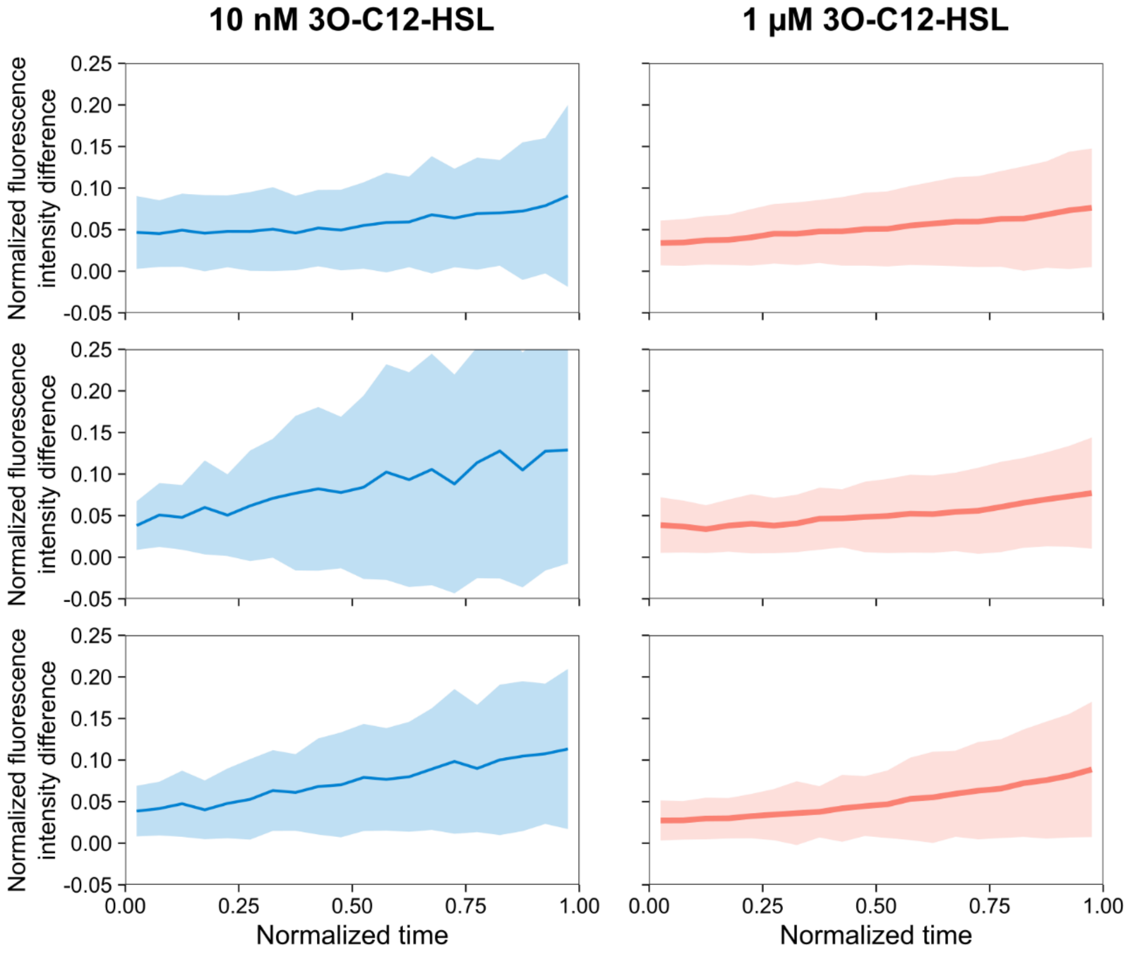

Supplementary Figure S12.

Experimental replica-based analysis of phenotypic traits considering cell lineage information in case of 10 nM (left panel) and 1 □M (right panel) signal molecule concentrations. a) The average normalized fluorescence intensity difference for pairs of cells concurrently present in a device as a function of their cell lineage distance. b) Probability of being in opposite QS state for pairs of cells concurrently present in a device as a function of their cell lineage distance. Bar graphs show the averages of the three biological replicates together with standard deviations.

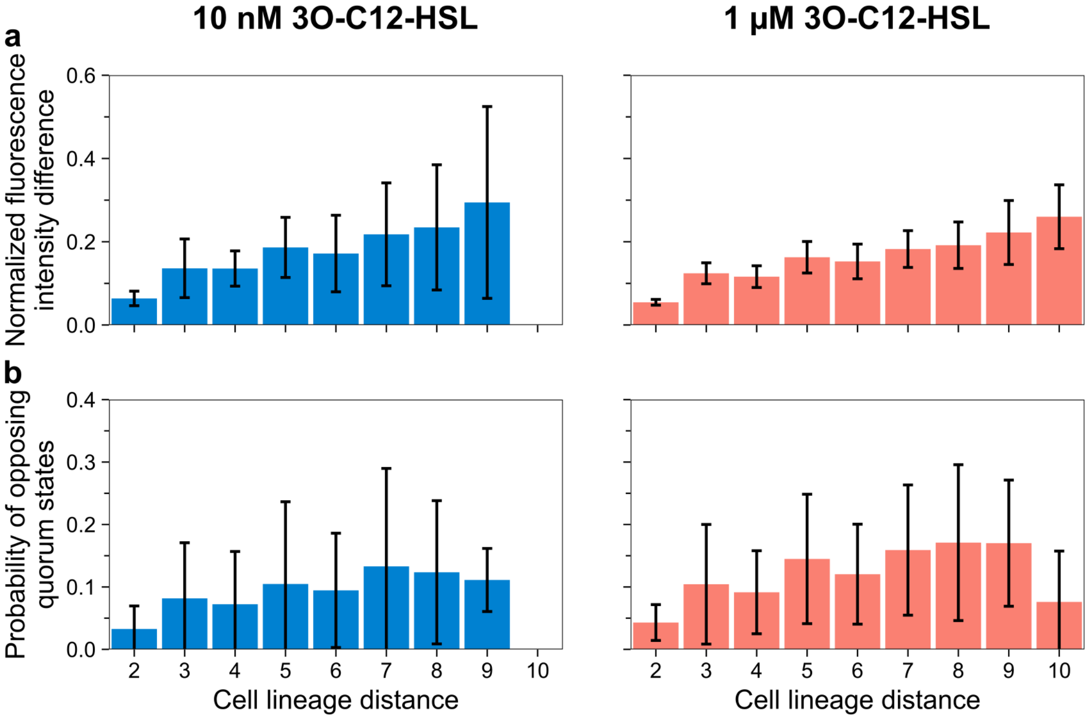

Supplementary Figure S13.

Number of cell pairs in CLD analysis and number of cells during the experiments with repeated signal pulses. a) Number of cell pairs in CLD analysis in case of 10 nM (left panel) and 1 □M (right panel) signal molecule concentrations. Criteria: at least 5 cell pairs. b) Number of cells (aggregated dataset) during the 44-hour long experiments (1 µM signal-on/off/on/off). c) Number of cell pairs in CLD analysis in case of the 44-hour long experiment (1 µM signal-on/off/on/off). Criteria for the analysis: at least 5 cell pairs at a time point.

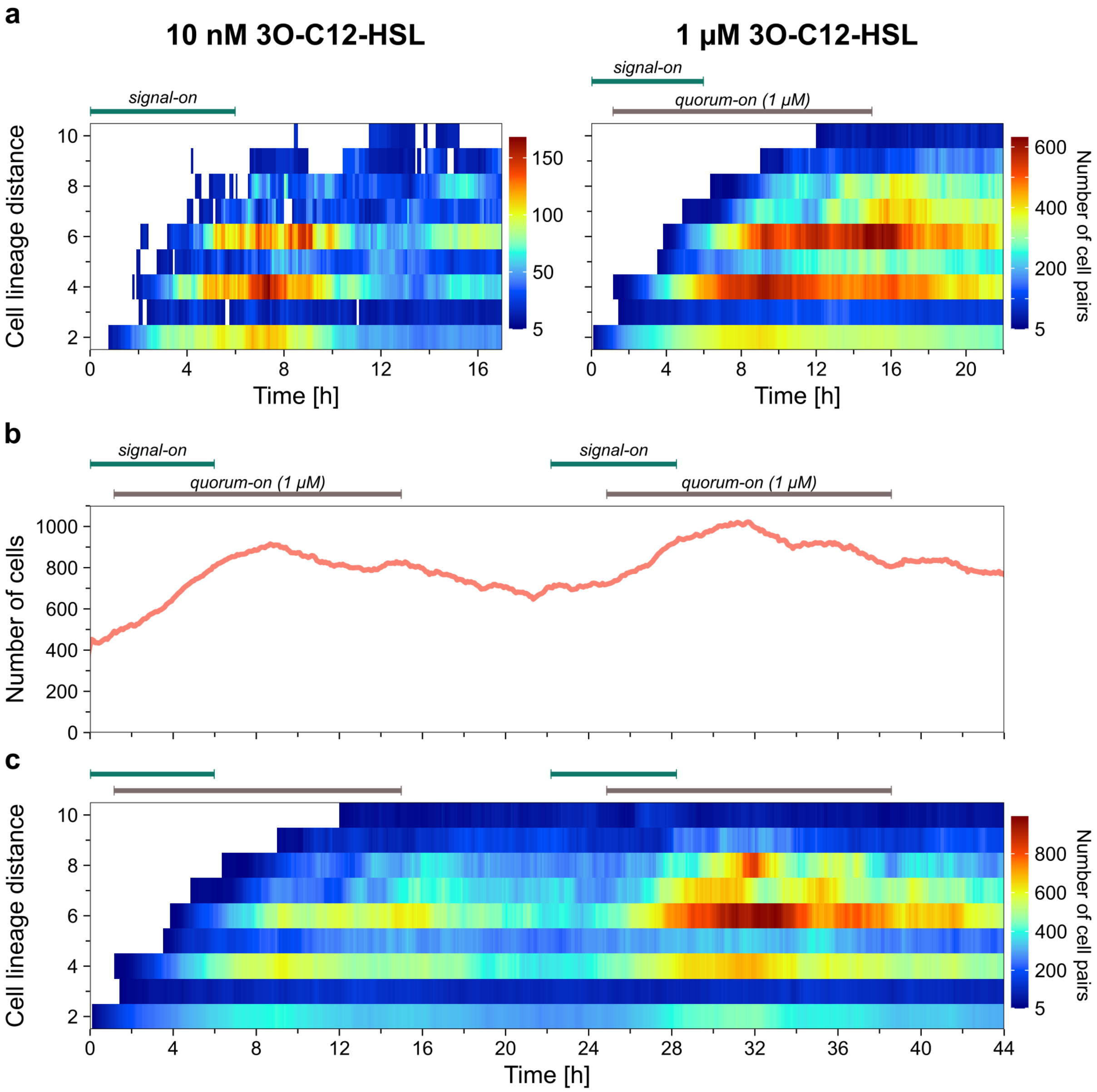

Supplementary Figure S14.

Average fluorescence intensity of signal-blind (Δ*lasR* Δ*rhlR*) and Δ*lasI* *P. aeruginosa* PUPa3 cultures with and without QS stimulation. Bacterial populations were incubated in signal-off and signal-on medium in a 96-well plate reader for eight hours (30 °C, continuous shaking; for further details, see the Supplementary Methods). The fluorescence intensity values were normalized with the measured optical density (at 600 nm). Six replicates were measured.

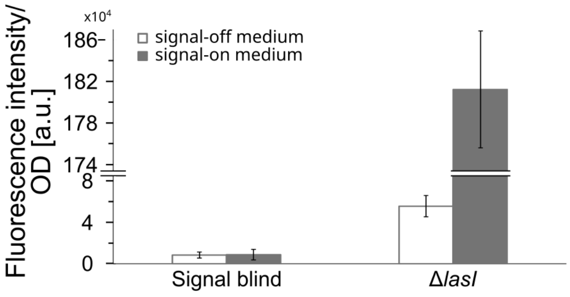

Supplementary Figure S15.

Segmentation and tracking of bacteria in the mother machine device using BACMMAN software. The segmentation and tracking of bacterial cells and divisions are presented in one microchannel over time. The image is part of the editable kymography generated by BACMMAN from the time series images of an experiment. Colored lines connect corresponding cells on subsequent frames.

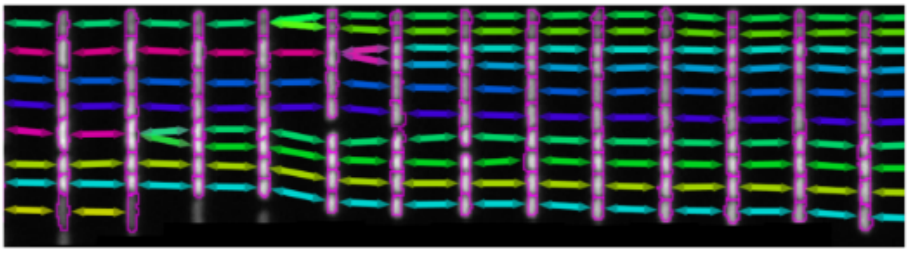

Supplementary Figure S16

Single-cell-level average fluorescence intensity distribution of *P. aeruginosa* PUPa3 Δ*lasI* strain without QS stimulation. Data from test tube bacteria cultures (24 hours without 3O-C12-HSL) (blue histogram, 7275 cells, for details, see Supplementary Methods) are compared to the first datapoint of the mother machine experiments right before QS induction with 1 □M 3O-C12-HSL (red histogram, 283 cells).

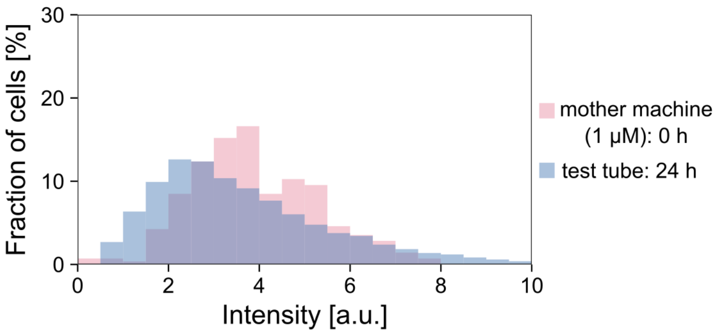

Supplement: Supplementary file 1 — Supplementary Information. [file 41598_2024_66706_MOESM1_ESM.pdf]
